# Parametric imaging of attenuation by optical coherence tomography: review of models, methods, and clinical translation

**DOI:** 10.1117/1.JBO.25.4.040901

**Published:** 2020-04-03

**Authors:** Peijun Gong, Mitra Almasian, Gijs van Soest, Daniel M. de Bruin, Ton G. van Leeuwen, David D. Sampson, Dirk J. Faber

**Affiliations:** a The University of Western Australia, Department of Electrical, Electronic and Computer Engineering, Optical+Biomedical Engineering Laboratory, Perth, Western Australia, Australia; b University of Amsterdam, Amsterdam University Medical Centers, Cancer Center Amsterdam, Amsterdam Cardiovascular Sciences, Department of Biomedical Engineering and Physics, Amsterdam, The Netherlands; c Erasmus MC, University Medical Center Rotterdam, Department of Cardiology, Rotterdam, The Netherlands; d University of Surrey, Surrey Biophotonics, Guildford, Surrey, United Kingdom

**Keywords:** optical coherence tomography, attenuation coefficient, single scattering, multiple scattering, dermatology, cardiology, oncology

## Abstract

**Significance:** Optical coherence tomography (OCT) provides cross-sectional and volumetric images of backscattering from biological tissue that reveal the tissue morphology. The strength of the scattering, characterized by an attenuation coefficient, represents an alternative and complementary tissue optical property, which can be characterized by parametric imaging of the OCT attenuation coefficient. Over the last 15 years, a multitude of studies have been reported seeking to advance methods to determine the OCT attenuation coefficient and developing them toward clinical applications.

**Aim:** Our review provides an overview of the main models and methods, their assumptions and applicability, together with a survey of preclinical and clinical demonstrations and their translation potential.

**Results:** The use of the attenuation coefficient, particularly when presented in the form of parametric *en face* images, is shown to be applicable in various medical fields. Most studies show the promise of the OCT attenuation coefficient in differentiating between tissues of clinical interest but vary widely in approach.

**Conclusions:** As a future step, a consensus on the model and method used for the determination of the attenuation coefficient is an important precursor to large-scale studies. With our review, we hope to provide a basis for discussion toward establishing this consensus.

## 1 Introduction

Optical coherence tomography (OCT) discriminates the backscattered light from a tissue sample based on the path length that the light has traveled, being exquisitely sensitive to light that has undergone one or a few scattering events.^1^ This extraordinary ability is achieved largely through coherence gating, augmented by confocal gating, and simultaneously rejects out-of-focus light and imposes a selected path length. Depth-resolved images of this backscattering in tissue can be obtained, *ex vivo* and *in vivo*, with a resolution commonly in the range 5 to 15  μm, although sub-1-μm resolution has been demonstrated.^2^ Currently, OCT is primarily used to visualize the morphology of tissue, which can be used to differentiate pathology in some circumstances.^3^ There is further clinical value, in addition to visualization, to use OCT to differentiate pathology based on the altered structure and organization not readily visible with conventional clinically available imaging techniques. The structure and organization of a tissue are reflected in its optical properties,^4^^,^^5^ and perhaps the most accessible such property in OCT is the attenuation coefficient, describing the extinction with depth of the detected OCT signal due to absorption and scattering. To measure the OCT attenuation coefficient ($\mu_{\mathrm{OCT}}$) and obtain diagnostic information from this measure, a model of the OCT signal and a model correlating $\mu_{\mathrm{OCT}}$ to the optical properties (absorption and scattering coefficient; Sec. 2), and ultimately to the tissue structure, must be developed (Sec. 3).^6^^–^^8^ Recently, with the common advent of volumetric OCT imaging, it has become feasible to produce two-dimensional (2-D) *en face*, and even three-dimensional (3-D) depth-resolved maps of $\mu_{\mathrm{OCT}}$, representing an example of the general class of parametric imaging.^9^ Consequently, this topic has generated increased interest in the literature.^10^^–^^12^

The onset and advance of diseases or injury are often accompanied by structural and functional changes in tissues. These changes can range from easily visible scars, to increased blood perfusion during inflammation (which may be observed as redness), to an increase of intracellular mitochondrial proliferation during the early stages of cancer development. A major difference lies in the length scales at which these changes occur. Whereas scars in skin are readily observed by visual inspection, the assessment of subcellular changes requires higher sensitivity and resolution. With such techniques for *in vivo* assessment not widely available, the current standard for early diagnosis is the excision of small tissue sections followed by histochemical staining and microscopic evaluation by a pathologist. For many applications on all length scales, OCT may provide a viable alternative that mitigates the drawbacks of histopathology: be it in terms of patient well-being, by enabling less invasive and more immediate diagnostic procedures, or in terms of economic cost, by reducing the number of unnecessary pathological assessments.

First and foremost, OCT provides high-resolution 3-D imaging of tissue structures. For example, OCT can quantify epithelial layer thickening (up to the point of disappearance of tissue layering) that is associated with increasing stage (growth) of cancer. Second, subresolution changes in tissue morphology during onset and progression of disease lead to changes in optical absorption and scattering properties of the tissue that can be assessed through quantitative measurement of the OCT signal decay with depth. These subresolution changes are not directly available to imaging, leading to poor contrasts in the tissue structures provided by conventional OCT. For tissue characterization relevant to such small-scale changes, quantitative measurement of the tissue attenuation forms an important complement to conventional OCT (Sec. 4).

Preclinical and clinical studies in a wide variety of medical fields, including dermatology and skin, in general, cardiology, and urology, have shown promising results on the use of $\mu_{\mathrm{OCT}}$ for tissue characterization.^9^^,^^13^^–^^19^ The associated literature presents multiple models and methods to determine $\mu_{\mathrm{OCT}}$ and to relate it to tissue optical properties. To advance the application of $\mu_{\mathrm{OCT}}$ for tissue characterization, a standardized and validated approach to obtain reliable values of $\mu_{\mathrm{OCT}}$, and to deal with issues such as tissue heterogeneity and the length scales on which this occurs, is needed. The aim of this review, then, is to present an overview of the models, methods, and applications of parametric imaging of attenuation by OCT and to discuss issues in the determination of $\mu_{\mathrm{OCT}}$ with the ultimate goal of establishing a unified basis for future clinical research on using $\mu_{\mathrm{OCT}}$. To this end, the review is divided from here on into four sections. In Sec. 2, the relationship between tissue optical properties and the OCT attenuation coefficient is discussed. In Sec. 3, commonly used models for the OCT signal are summarized. In Sec. 4, an overview of potential preclinical applications and clinical translation of $\mu_{\mathrm{OCT}}$ is given, accompanied with a summary of the reported $\mu_{\mathrm{OCT}}$ values. Finally, in Sec. 5, the limitations of the models and methods, together with clinical challenges and future perspectives, are discussed.

## 2 Tissue Optical Properties

Absorption and scattering, the two components of attenuation, fundamentally arise from (spatial variations in) the complex refractive index of tissue m(r)=n(r)+iκ(r). The local absorption coefficient is directly proportional to the imaginary part of the complex refractive index through $\mu_{a}(r)=2k\kappa (r)$, where k=2π/λ is the wavenumber; and λ is the wavelength. Gradients in the real refractive index n(r) redirect light by refraction on a microscopic scale and, thus, redistribute its propagation direction, determining the local scattering coefficient and phase function. Absorption directly reduces the light intensity by converting it into other forms of energy. It is parameterized by the absorption coefficient $\mu_{a}$, which, for a homogeneous distribution of absorbers, is the product of the density and absorption cross section of the absorbing particles. The wavelength-dependent absorption spectrum of tissue is determined by the presence of various chromophores in tissue, where hemoglobin, melanin, and water are dominant. However, in order to achieve maximum imaging depths, OCT generally operates in near-infrared spectral regions where the absorption by these chromophores is low. For this reason, $\mu_{\mathrm{OCT}}$ is dominated by attenuation due to scattering at the commonly used wavelengths (800 and 1300 nm as shown in Sec. 4) for OCT attenuation coefficient analysis. Previous studies have shown approximately 10 times higher scattering than absorption in the near-infrared ranges typically used for OCT.^20^^,^^21^ Thus, the effects of absorption, which are notably present in OCT using visible wavelengths,^22^ spectroscopic OCT,^23^^,^^24^ and low-coherence spectroscopy,^25^ do not play a role in the research on the OCT attenuation coefficient reviewed here and will not be discussed further.

Analogous to absorption, the scattering strength is parameterized by the scattering coefficient $\mu_{s}$, which, for low particle densities, is the product of the particle density and scattering cross section. The scattering coefficient depends both on the wavelength and the scatterer dimensions. In elastic scattering, which is relevant for OCT, no energy conversion takes place, but scattered light is spatially redirected and so the intensity of an incident wave is diminished. The scattering phase function describes this process of angular redistribution of energy. It is often convenient to parameterize the phase function in terms of Legendre moments, the first of which is called the scattering anisotropy g. It physically corresponds to the average cosine of the scattering angle (g=1, thus, implies all light is scattered in the forward direction). The phase function and scattering anisotropy are dependent on wavelength and scatterer dimensions as well. In general, large particles (with respect to wavelength) will scatter more strongly in the forward direction.

The absorption and scattering coefficients are formally defined in terms of interaction probability per unit path length. Their sum is the attenuation coefficient, $\mu_{t}=\mu_{a}+\mu_{s}$, which describes the decay of the incident light due to the tissue optical properties. In contrast, the OCT attenuation coefficient, $\mu_{\mathrm{OCT}}$, parameterizes the loss of OCT signal with depth, caused by absorption and scattering, when this loss is modeled as a single exponential decay in the form of Beer’s law, $\propto \exp(-\mu_{\mathrm{OCT}}z)$. This simple parameterization absorbs most of the complexity, but sacrifices a direct relation to tissue optical properties (e.g., anisotropy, g; $\mu_{a}$; $\mu_{s}$) for the sake of robustness and uniqueness of the measurement. In addition, OCT system properties, such as the confocal point spread function (CPSF) and sensitivity roll-off (for Fourier-domain OCT systems) also cause a depth-dependent response. We will discuss in Sec. 3 how various signal models summarize tissue optical properties in a single parameter, $\mu_{\mathrm{OCT}}$. Modeling the connection between tissue structure, optical properties, and measured quantities remains a formidable challenge, which we will not resolve in this review. Most research discussed here will correlate tissue type and structure (through the gold standard of histopathology) to the measured OCT attenuation coefficient.

## 3 Models of the OCT Signal

**Fig. 1:**
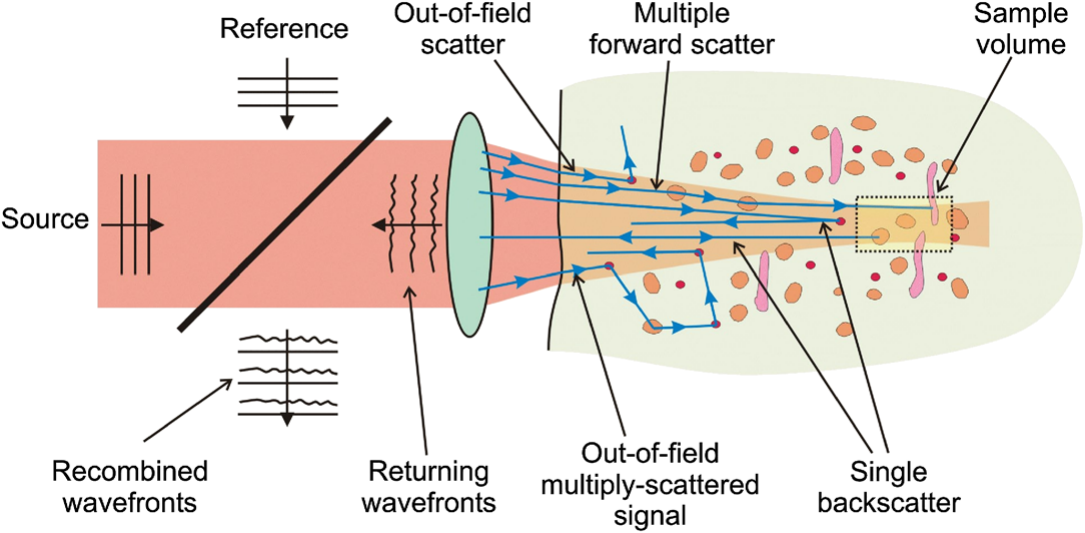
Propagation of a focused OCT beam and ray representation of scattering in the sample.^27^

In OCT, the field in the sample arm results from electromagnetic scattering of broadband light in tissue, a complex dielectric. Because of the variations of the complex refractive index m(r) in tissue, and because of the many length scales at which this variability contributes to the scattered field,^26^ it is generally impossible and almost certainly impractical to strive for an exact inversion of Maxwell’s equations, solving them for m(r). As the field propagates, energy is removed from the incident beam while backward scattering feeds the returning field, as shown in Fig. 1; the attenuation coefficient $\mu_{\mathrm{OCT}}$ is a single parameter to quantify the strength of this interaction of light and tissue. Extraction of the $\mu_{\mathrm{OCT}}$ from the acquired data requires a quantitative model for the OCT signal that can be applied to the measurement.

A number of different models have been proposed to describe the OCT signal. In order of increasing complexity and the number of free parameters, these models may be summarized as “single-scattering,” “multiple-scattering” (including stochastic methods such as Monte Carlo), and “full electromagnetic wave modeling.” Among them, the single-scattering model (Sec. 3.1) is most commonly used, whereas others (Sec. 3.2) have been applied to a smaller number of samples or are still in the early stages of development. All of these models need to be calibrated with system-specific parameters for practical applications.

### 3.1 Single-Scattering Model

The simplest models for the OCT signal rely on the first-order Born approximation; they assume that the incident beam propagates in the forward direction, attenuated by absorption and scattering according to Beer’s law, until a backward scattering event reflects the light back toward the source. Any detected light from the sample arm has interacted with the sample only in a single-scattering event. For plane wave illumination, homogeneous optical scattering, and in the absence of noise and any instrumental effects, the mean OCT signal may be written as^28^

$$\langle A(z)\rangle \propto A_{0}\;\exp(-\mu_{\mathrm{OCT}}\cdot z),$$ (1)

where A(z) is the OCT signal amplitude, $A_{0}$ is the amplitude at z=0 (the tissue boundary), and $\mu_{\mathrm{OCT}}$ is the attenuation coefficient. Brackets ⟨·⟩ denote the average over different spatial realizations of the complex refractive index m(r).

One subtlety may be noted: in Beer’s law, the absorption coefficient $\mu_{a}$ and scattering coefficient $\mu_{s}$ govern the decay of the intensity I(z), not of the field amplitude described in Eq. (1). The attenuating medium is, however, traversed twice since the signal travels from the source to a depth z and then back toward the detector, and the path length is equal to 2z. Thus, the detected signal is $\left\langle I(z)\rangle =I_{0}\;\exp(-2\mu_{\mathrm{OCT}}z\right)$ but since in the absence of noise $\langle A(z)\rangle \propto \sqrt{\left\langle I(z)\right\rangle }$, the factor of 2 in the exponent is canceled in Eq. (1). In Fourier-domain OCT, the spatial signal is obtained through Fourier transform. For optimum processing speed, in most applications, the fast-Fourier transform (FFT) algorithm is used, after resampling of the spectral interferogram S(k) onto a uniform wavenumber (k) basis. The outcome of this procedure is the OCT amplitude versus depth as given by Eq. (1). Alternatively, the power spectral density or ${\left|\mathrm{FFT}\right|}^{2}$ may be used, which would yield the OCT intensity versus depth.

The models discussed here aim to quantitatively describe the OCT signal in terms of the tissue attenuation, $\mu_{t}=\mu_{a}+\mu_{s}$, a number that is based on the assumption of single scattering. However, even in the presence of multiple scattering (Sec. 3.2), the part of the signal decay caused by absorption and scattering is often adequately modeled as a single exponential decay, albeit with a decay coefficient $\mu_{\mathrm{OCT}}<\mu_{t}$ (because multiple scattering causes more light to be detected than expected based on the single-scattering model). Without additional controlled experiments, for instance at different scatterer concentrations, it may not be possible to say with certainty that a measurement was done in the single-scattering regime. Thus, the adoption of $\mu_{\mathrm{OCT}}$ allows us to describe tissue attenuation as measured by OCT as an effective parameter that does not require an estimate of the relative weight of single and multiple scattering contributions.

#### 3.1.1 Practical application of the single-exponential decay model

Already when introducing this model for the OCT signal, Schmitt et al.^1^ realized that Eq. (1) needs to be modified for finite numerical aperture (NA). They introduced a correction for the divergence of the sample beam and used this to quantify the attenuation and backscattering coefficients of weakly scattering microsphere suspensions. In addition, for Fourier-domain OCT, the system sensitivity decreases with depth from the zero-delay point depending on the sampling of the wavenumber axis. A constant factor α<1 describes the coupling efficiency of the input amplitude $A_{0}$ to the OCT system, which is redirected back toward the detector with a power-backscattering coefficient $\mu_{b,\mathrm{NA}}$. With these factors taken into account, including noise, Eq. (1) becomes

$$\langle A(z)\rangle =\alpha \cdot A_{0}\cdot t(z-z_{f})\cdot h(z-z_{0})\cdot \sqrt{\mu_{b,\mathrm{NA}}}\;\exp(-\mu_{\mathrm{OCT}}z)+\text{noise}.$$ (2)

Here, the backscattering coefficient, $\mu_{b,\mathrm{NA}}$, depends on the NA of the system since that determines the collection angle. Coordinate z is the geometrical distance from the tissue boundary, into the tissue. The factor $t(z-z_{f})$ is the CPSF, which is derived from the beam divergence correction factor^1^ to yield the following expression for a Gaussian beam:^29^

$$t(z-z_{f})=\frac{1}{\sqrt{{\left(\frac{z-z_{f}}{2nz_{R}}\right)}^{2}+1}},$$ (3)

where $z_{f}$ is the geometrical depth location of the focus relative to the tissue boundary, $z_{R}$ is the Rayleigh length of the Gaussian beam incident on the sample, and n is the average refractive index of the medium along the beam. The factor of 2 in $2nz_{R}$ takes into account the increase in $z_{R}$ for a diffuse reflector compared to specular reflector.^29^^,^^30^

The sensitivity roll-off of the system is described by $h(z-z_{0})$, where $z_{0}$ is the distance between the zero-delay position of the interferometer and the tissue boundary. The finite sampling density of the interference fringes and the finite resolution of the frequency scan together reduce the signal far from the zero-delay position. The former derives from the detector pixel width in spectral-domain OCT, and from the detector integration time in swept-source OCT, and is described by a sinc function. The latter factor is the spectrometer optical resolution in spectral-domain OCT (spot size in the spectrometer), or the instantaneous linewidth in swept-source OCT, and can be modeled as a Gaussian function. Combining resolution and sampling dependencies, $h(z-z_{0})$ is expressed as^31^

$$h(z-z_{0})=|\mathrm{sinc}[\frac{\pi (z-z_{0})}{2z_{D}}]|\exp\{-\frac{\pi^{2}s^{2}}{16\;\ln(2)}{\left[\frac{\left(z-z_{0}\right)}{z_{D}}\right]}^{2}\}.$$ (4)

**Fig. 2:**
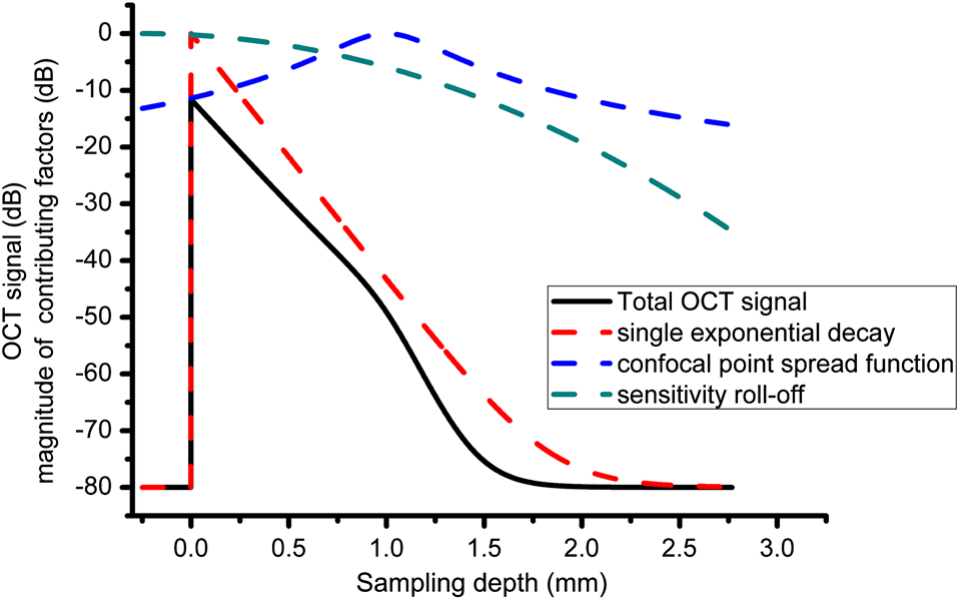
Simulated average A-scan ⟨A(z)⟩ according to Eq. (2) (black curve) and the contribution of the individual terms, including single exponential decay (red dash), CPSF t(z) (blue dash), and sensitivity roll-off h(z) (green dash). Parameters for the simulation are: $z_{0}=0.25\;\mathrm{mm}$; $\mu_{s}=5\;{\mathrm{mm}}^{-1}$; refractive index n=1.4; noise floor at −80  dB; Rayleigh length $z_{R}=100\;\mu m$; focus location $z_{f}=1\;\mathrm{mm}$; center wavelength and spectral sampling increment are λ=1300  nm and Δλ=0.1  nm, respectively, giving a maximum imaging depth of $z_{D}=3\;\mathrm{mm}$; s=2.

Here, $z_{D}=\lambda^{2}/4n\Delta \lambda$ is the maximum imaging depth of a system with sampling pitch Δλ at center wavelength λ and at average group refractive index n of the medium, measured with respect to zero delay, and s is the ratio of spectral resolution to the sampling pitch.^32^^,^^33^ Since spectral resolution and sampling pitch are usually finer in swept source systems, $h(z-z_{0})$ is flatter for swept-source systems compared to spectral-domain systems, resulting in a higher signal-to-noise ratio (SNR) at greater depths. Examples of the confocal PSF and sensitivity roll-off from a swept-source OCT scanner are shown in Fig. 2.

Measurement of the attenuation coefficient requires quantitative modeling of the instrumental response function, CPSF, and sensitivity roll-off, according to Eqs. (3) and (4). If the detailed optical design parameters (specifically, the focal depth $z_{f}$ and Rayleigh length $z_{R}$ of the imaging optics, and resolution and sampling of the frequency scan) are unknown, these functions can be experimentally determined from the measurement of a reflector versus depth, and a knife-edge measurement may be used to yield the Gaussian beam parameters.^32^^,^^34^ Alternatively, a very weakly scattering calibration sample may be used to determine the CPSF and sensitivity roll-off function by substituting Eqs. (3) and (4) into Eq. (2) and then fitting it to the OCT signal amplitude, with s, $z_{f}$, and $z_{R}$ as free parameters and setting $\mu_{\mathrm{OCT}}=0$. Another approach based on an OCT measurement of a very weakly scattering calibration sample is by first subtracting the mean noise level (typically estimated in a deep region with only noise present) included in Eq. (2) and then assuming $\mu_{\mathrm{OCT}}=0$ so that the signal of the calibration sample only comprises $\alpha \cdot A_{0}\cdot t(z-z_{f})\cdot h(z-z_{0})\cdot \sqrt{\mu_{b,\mathrm{NA}}}$. The signal from a highly scattering sample, after removing the noise level similarly, can then be divided by the calibration signal, canceling the $\alpha \cdot A_{0}\cdot t(z-z_{f})\cdot h(z-z_{0})$, directly yielding the data for attenuation analysis.^17^ For example, Gong et al.^35^ used a solution of 0.5-μm-diameter polystyrene microspheres with an estimated attenuation coefficient of $0.1\;{\mathrm{mm}}^{-1}$. As the CPSF is influenced by the refractive index of the sample, it is desirable for the calibration sample to have a refractive index similar to the samples to be studied.

#### 3.1.2 Fitting method

**Fig. 3:**
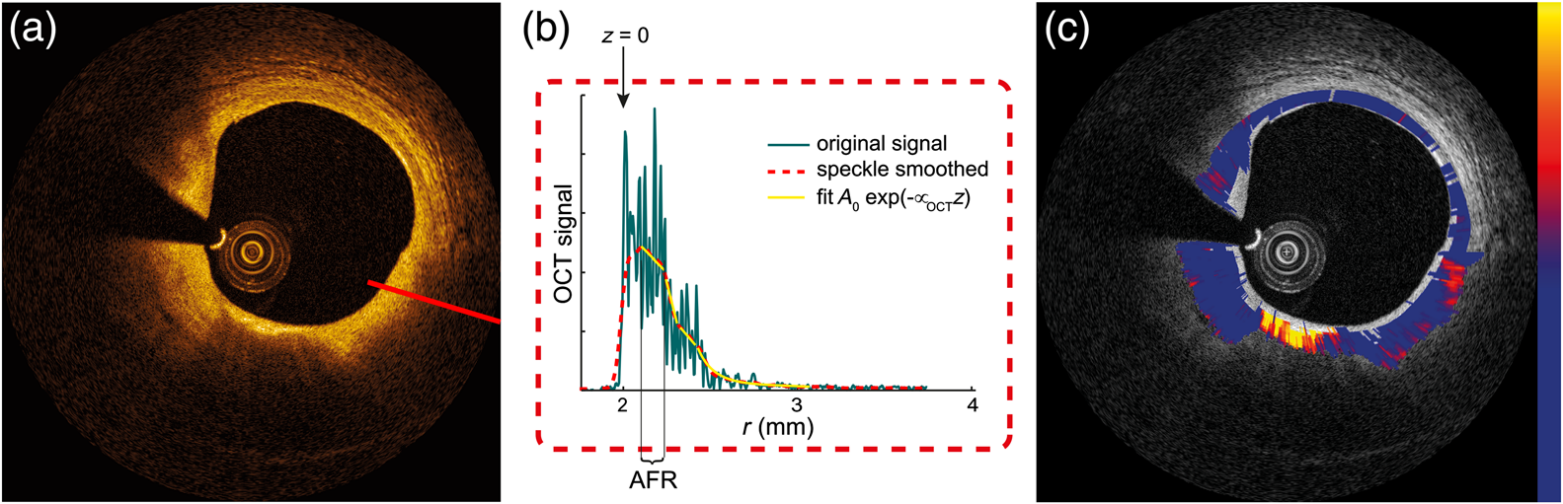
Procedure of fitting to retrieve the attenuation coefficient. One A-scan in (b) is extracted from an intravascular OCT image in (a) along the red line, speckle smoothed and fitted in subsequent windows. The retrieved $\mu_{\mathrm{OCT}}$ is plotted as an overlay on the grayscale image in (c). Colormap: 0 to $12\;{\mathrm{mm}}^{-1}$.

Nonlinear least squares curve fitting, preferably applied to the amplitude data, is the most straightforward approach for obtaining $\mu_{\mathrm{OCT}}$ using the single-scattering model: by varying the free parameters $A_{0}$ and $\mu_{\mathrm{OCT}}$ while fixing the other parameters to optimize the fit of Eq. (2). Note that if the noise is not corrected or cannot be ignored, squaring the OCT amplitude induces cross terms that can influence the fitted attenuation coefficient. Alternatively, a linear fit to the logarithm of the OCT data can be performed after subtraction of the logarithm of the CPSF, sensitivity roll-off, and noise from the data. Any fit method requires a region of interest (ROI) or window selection of axial fitting range (AFR), as shown in Fig. 3, which can be done manually or automatically. We note, however, that for most applications, manual selection would be too labor-intensive in any eventual routine use. The analysis is performed under the assumption that the tissue optical properties are homogeneous within the window, so the axial resolution of the attenuation measurement is limited by this window size (i.e., AFR). This assumption may be verified by uncertainty and goodness-of-fit estimates (e.g., $R^{2}$ and residue) used to assess fit quality. Challenges within the fitting method are the influence of speckle on the goodness of the fit, AFR selection, and noise-level selection. Lateral and/or axial averaging can reduce the variability due to speckle in the A-scan data prior to fitting. By averaging, the resolution of the measured $\mu_{\mathrm{OCT}}$ is decreased, the suitability of which must be considered against the needs of the application.

Performing linear fits on logarithmically compressed data is computationally more efficient, but the procedure is sensitive to underestimation of large attenuation coefficients in the presence of noise, depending on the window length. Yuan et al.^36^ showed that values of $\mu_{\mathrm{OCT}}$ of 5 and $7\;{\mathrm{mm}}^{-1}$ were underestimated in linear fits for ROIs larger than 800 and 600  μm, respectively. A shorter window means that stronger attenuation can be characterized without noise compromising the analysis. Low amplitudes at the end (greater z) of the ROI, which are more strongly affected by noise, have a larger weight in the compressed than in the noncompressed data. This issue may be readily dealt with by weighting the data point by the SNR, as has been used in optical coherence elastography.^37^

The range in which the fit can be considered valid can be assisted by a comparison between the modeled signal decay within the ROI, compared to the noise level of the OCT system; the range typically is in the order of a few times the attenuation length (about 4 times in the example cited above).^36^ Multiple fits with a small depth variation of the ROI may yield an average and standard deviation of $\mu_{\mathrm{OCT}}$; in this way, the influence of the ROI selection on the obtained value of $\mu_{\mathrm{OCT}}$ can be taken into account.^28^ Heterogeneity in the tissue and buildup of multiple scattering can be mitigated by adaptively choosing a fitting ROI and analyzing each A-scan in sections leading to 2-D parametric imaging of tissue properties.^16^ Fitting of very thin tissue layers (e.g., retinal layers) or tissue layers close to the surface (e.g., the epidermis) is challenging because there are insufficient sample points for a reliable fit within a homogeneous layer.^38^

#### 3.1.3 Depth-resolved method

An interesting depth-resolved method was proposed by Vermeer et al.^38^ for pixel-by-pixel determination of $\mu_{\mathrm{OCT}}$ inspired by attenuation compensation in ultrasound data.^39^ It does not require a fit window and, therefore, retains the OCT resolution in the attenuation image. Based on an integral formulation of the intensity [and not amplitude as in Eq. (1)] in the single-scattering model, $\mu_{\mathrm{OCT}}$ is calculated based on two main assumptions: (1) all the light is extinguished within the OCT image depth range; and (2) the backscattered light is a fixed fraction of the attenuation coefficient, i.e., the ratio of $\mu_{b,\mathrm{NA}}$ and $\mu_{\mathrm{OCT}}$ is constant. Assuming a constant intensity over a pixel, the attenuation is expressed as

$$\mu_{\mathrm{OCT}}[i]=\frac{1}{2\Delta }\;\log(1+\frac{I[i]}{\sum_{i+1}^{\infty }I[i]}),$$ (5)

where i is the i’th pixel along an A-scan and I[i] is the intensity of the signal at the i’th pixel. This equation can be simplified by applying a first-order linearization of log(1+x) around x=0 to give

$$\mu_{\mathrm{OCT}}[i]\approx \frac{I[i]}{2\Delta \sum_{i+1}^{\infty }I[i]}.$$ (6)

It is noteworthy that the validity of the sum to infinity in Eq. (6) requires that the values of the OCT signal in the last pixels in the image should be negligible, meeting the first assumption above. In the original formulation^38^ and subsequent applications^40^ of the depth-resolved attenuation coefficient analysis, the CPSF and sensitivity roll-off in depth were not taken into account. Smith et al.^41^ introduced these corrections, including for noise. The depth-resolved method was validated on homogeneous and layered phantoms by Vermeer et al.^38^ and showed promising results for estimating attenuation coefficients with a higher axial resolution. As pointed out above, though, the method relies on the assumptions of complete extinction in an A-scan and a fixed ratio between $\mu_{\mathrm{OCT}}$ and $\mu_{b,\mathrm{NA}}$. The former becomes more problematic for pixels toward the end of the A-scan and when prominent multiple-scattering background is present, which can be eliminated by carefully choosing a cutoff constant.^40^^,^^42^ The latter assumption does not hold in the case of absorption or for scatterers with a strongly structured angular scattering cross section, such as Mie scatterers. Since $\mu_{\mathrm{OCT}}$ is directly proportional to I[i] in Eq. (6), the analyzed attenuation contains speckle. This artifact may be dealt with by conventional methods such as local averaging or median filtering, which will then lower the resolution. Validation studies on tissue are still scarce, so the impact of the assumptions and necessary postprocessing on the accuracy of the extracted attenuation coefficients, and their utility for tissue classification, is not yet clear.

Alternatively, Yuan et al.^36^ proposed a distinct frequency-domain method, aided by Fourier transformation, to extract the attenuation coefficients. The method was compared to the fitting method, showing robust performance and fast computation. It is a potentially powerful alternative to the fitting and depth-resolved methods, the merits of which may become clear with wider adoption in future.

### 3.2 Multiple Scattering

In addition to singly scattered light, multiply scattered light that matches the detected optical path length set by the reference delay contributes to the OCT signal.^43^^–^^46^ In highly forward scattering tissues such as blood, multiple scattering can be expected.^47^^–^^49^ The contribution of multiply scattered light leads to a lower resolution and introduces a signal additional to that of singly scattered light. The contribution from multiply scattered light increases for (1) larger depths,^50^ (2) samples with stronger forward scattering,^28^ (3) samples with higher scattering coefficients,^28^ and (4) lower NAs.^50^

**Fig. 4:**
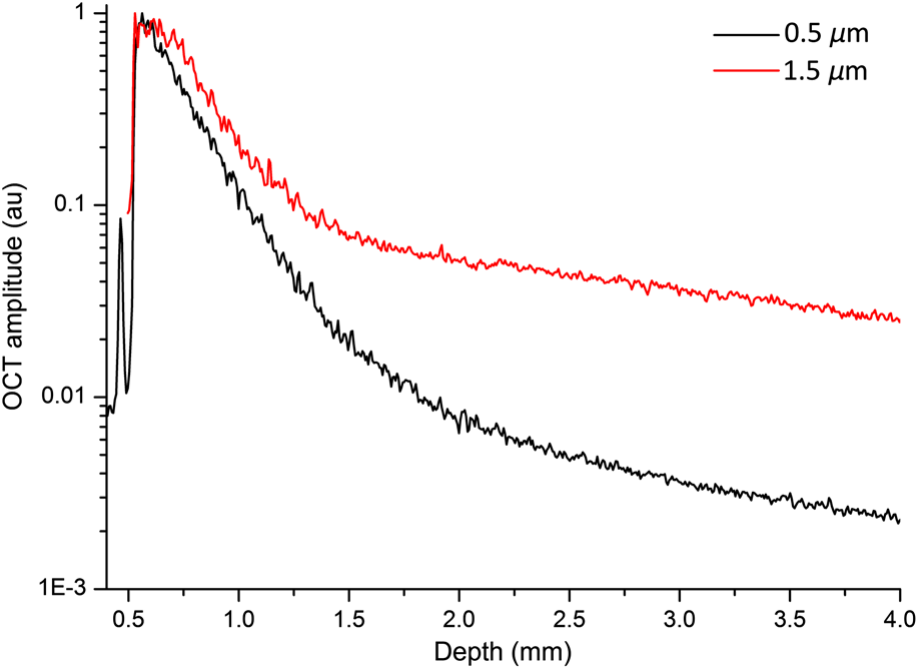
Average OCT amplitude for samples of pure silica beads in water. The 0.5-μm beads (black) with scattering coefficient of $5\;{\mathrm{mm}}^{-1}$ and anisotropy factor of 0.1 and 1.5-μm beads (red) with scattering coefficient of $5\;{\mathrm{mm}}^{-1}$ and anisotropy factor of 0.9. OCT data were collected using a swept-source 1300-nm system with a 150-mm-focal length detection lens.

Figure 4 shows the difference in OCT signal between an isotropic and anisotropic (forward scattering) sample in order to demonstrate the contribution of multiply scattered light. The two samples comprise silica beads with diameter of, respectively, 0.5 (in black as the isotropic sample) and 1.5  μm (in red as the anisotropic sample). They have the same attenuation coefficient estimated by Mie simulation and present the same linear decay (i.e., slope) in the logarithmic OCT signal in the single-scattering regime. Multiple scattering lifts up the linear decay at large depths with the large particles exhibiting the contribution by multiply scattered light from a much shallower depth than the small particles and, thus, generating more significant deviation from the single-scattering model. Faber et al.^30^ have shown that, for scattering media with $\mu_{s}<6\;{\mathrm{mm}}^{-1}$, the single-scattering model-based $\mu_{\mathrm{OCT}}$ gives a good estimate of $\mu_{s}$.^51^^,^^52^ Experiments on samples with controlled optical properties show that multiple scattering starts to contribute significantly to the OCT signal for samples with $\mu_{s}>10\;{\mathrm{mm}}^{-1}$ or g>0.8.^28^

To date, there are three main approaches to take into account for multiple scattering in OCT: probabilistically, with Monte Carlo simulations;^53^^,^^54^ and analytically, with the extended Huygens–Fresnel (EHF) model for OCT^45^^,^^55^^,^^56^ or *ab initio* full-wave simulations based on Maxwell’s equations.^57^^–^^59^ We briefly discuss each below.

#### 3.2.1 Monte Carlo simulation

Monte Carlo simulation is a probabilistic approach to simulate the scattering trajectory of photons in the sample. The simulation tracks the trajectory of photons in the sample arm and outputs the photon count and corresponding path lengths. An assumption for the phase function of the sample is needed as an input to the simulation, and restrictions on photon count and trajectory are required to create time-efficient simulations. Multiple studies have been done on Monte-Carlo-based simulations of the OCT signal.^46^^,^^60^^,^^61^ Jacques et al.^53^ applied Monte Carlo simulation to derive a general equation to correct the OCT attenuation coefficient for the contribution of multiple scattering to determine tissue optical properties, including $\mu_{s}$ and g. This approach was applied in subsequent studies by Levitz et al.^54^ to study the growth of collagen gels.

In general, Monte Carlo simulations do not model the interference of the reference with the sample light explicitly. Karamata et al.^43^^,^^44^ combined their analytical model, in which the coherence was taken into account, with Monte Carlo simulations to account for both singly and multiply scattered light. A limitation of Monte Carlo simulations is that the results obtained depend on the specific chosen input parameters, such as the system-specific optical geometry, and the phase function, which is generally not well known for tissue and, indeed, unlikely to be constant across the simulated region.^62^ Monte Carlo approaches are useful for forward modeling, but their probabilistic output cannot be straightforwardly inverted for analysis of experimental data.

#### 3.2.2 Extended Huygens–Fresnel model

The EHF model for OCT was introduced by Schmitt and Knüttel^45^ and elaborated by Thrane et al.^55^^,^^56^ The model assumes the paraxial approximation [i.e., sin(θ)≈θ, where θ is the angle of the scattered wavevector relative to the incident wavevector] and the theory is applicable to samples with g>0.7. The mean OCT intensity is expressed in three terms: (1) the singly backscattered field, (2) the multiply (forward) scattered field, and (3) the coherent cross-term between these two fields. The expression for the mean squared OCT amplitude (which is equal to OCT intensity) is

$$\langle A^{2}(z)\rangle \propto \frac{1}{w_{H}^{2}(z)}\{\exp(-2\mu_{s}z)+\frac{4\;\exp(-\mu_{s}z)[1-\exp(-\mu_{s}z)]}{1+\frac{w_{S}^{2}(z)}{w_{H}^{2}(z)}}+{\left[1-\exp(-\mu_{s}z)\right]}^{2}\frac{w_{H}^{2}(z)}{w_{S}^{2}(z)}\},$$ (7)

where

$$w_{H}^{2}(z)=\;w_{0}^{2}[{\left(\frac{z-z_{f}}{2nz_{R}}\right)}^{2}+1],$$ (8)

$$w_{S}^{2}(z)=w_{H}^{2}(z)+\frac{1}{3}(\mu_{s}z)\theta_{\mathrm{RMS}}^{2}{\left(z/n\right)}^{2},$$ (9)

and z is the depth coordinate in tissue measured from the sample boundary at $z_{0}$. Equation (8) is the expression for the local beam waist in the absence of forward scattering, $w_{H}$. Here, $w_{0}$ is the beam waist at the focus in air. The term $\theta_{\mathrm{RMS}}$ is the root mean square of the average scattering angle and related to the scattering anisotropy through $\theta_{\mathrm{RMS}}\approx \sqrt{2(1-g)}$. The factor of 2 in front of the Rayleigh length of the beam is introduced to account for the doubling of the Rayleigh length for diffuse reflection, as described earlier.^30^ Equation (9) is the expression for the local beam waist in the presence of multiple forward scattering, $w_{S}$.^56^ Based on the EHF model, multiply scattered light influences the OCT signal at all depths. Assuming highly forward scattering media, the EHF model can be fitted to the OCT data using Eq. (7) to obtain tissue optical properties, including $\mu_{s}$ and $\theta_{\mathrm{RMS}}$. This is in contrast with the single-scattering formalism, which absorbs the effects of multiple scattering in the effective attenuation coefficient $\mu_{\mathrm{OCT}}$.

A limitation of the EHF model is that $\theta_{\mathrm{RMS}}$ and $\mu_{s}$ are codependent parameters, which means that a change in $\theta_{\mathrm{RMS}}$ can be compensated with a change in $\mu_{s}$ without any change in the outcome of the fit statistics.^30^ *A priori* knowledge of $\theta_{\mathrm{RMS}}$ or $\mu_{s}$ of the sample can be used to restrict the fit.^63^ Alternatively, the EHF model is used with *a priori* knowledge of $\theta_{\mathrm{RMS}}$ and $\mu_{s}$ for controlled silica bead samples to simulate the OCT signal, in order to estimate the contribution of multiple scattering to the single-scattering model-obtained $\mu_{\mathrm{OCT}}$. The model-based estimations were in good agreement with the experimental data for a large range of scattering and anisotropy values in silica bead samples.^28^^,^^63^ An absorption term was recently introduced in the EHF model to measure not only the scattering coefficient and anisotropy but also the absorption coefficient, which is usually assumed negligible for OCT wavelengths.^64^ However, further validation of such absorption coefficient measurement is still needed.

#### 3.2.3 Modeling of the OCT signal with Maxwell’s equations

A full-wave mathematical model of OCT image formation, based on Maxwell’s equations, has been developed by Munro et al.^57^^–^^59^ Using this model, 2-D and 3-D OCT images can be simulated. Compared to the above-mentioned models, the Maxwell’s equations-based model does not need to assume the first-order Born approximation or to consider an ensemble average of the scattering particles. This full-wave approach could, in general, allow modeling of the OCT signal for a variety of system configurations, beam geometries (e.g., Gaussian or Bessel), and (subresolution) sample parameters without making any approximations. The refractive index distribution of the sample is used as an input parameter, which works well for controlled phantoms; however, at the moment, is not generally known for biological tissue, and often not even in statistical terms. Full-field models are very computationally expensive to run, especially for high NAs, which limits the volume and complexity of the sample to be analyzed.^58^ Efficiency improvements can be achieved by precomputing scatterer microstructure,^65^ and by using analytical solutions of Maxwell’s equations for specific shapes, such as scattering from cylinders.^66^^,^^67^ These models remain challenging to implement and use because of their complexity and computational expense. With further development and the increase in computing power, they offer great potential to explore the links between microscopic structure and macroscopic parameters in quantitative imaging of tissue optics and validate the accuracy of parametric models, such as single scattering or EHF.

### 3.3 Summary of Models

In summary, a variety of models for the OCT signal have been proposed in the literature, ranging in complexity from a single exponential fit to a full-wave mathematical model based on Maxwell’s equations. The simplest models lack consideration of system parameters and the contribution of multiply scattered light. However, as models increase in complexity, more input parameters are required. The EHF model requires codependent sample-related parameters, which cannot be determined independently without *a priori* knowledge of $\mu_{s}$ or g. For Monte-Carlo-based estimations, the same problem arises, for which the scattering phase function has to be assumed. Although multiple models for the OCT signal have been studied in the literature, the most frequently applied one remains the single exponential model, relying upon the predominance of single scattering, such that the first-order Born approximation is valid. The single exponential model, together with accurate correction for system parameters, provides a valid approach without the mutual dependence of the fit parameters. Alternatively, the depth-resolved method can be applied to estimate $\mu_{\mathrm{OCT}}$ per pixel in depth, avoiding assumptions of tissue homogeneity in the axial direction. Although more work is undoubtedly needed to establish the level of model sophistication required, it is likely that meaningful results require one to be in an image-forming regime, and that image formation likely requires the single scattering assumption to be valid. Once beyond this depth, the dominance of multiple scattering may mean that more sophisticated models do not bear fruit.

## 4 Applications of OCT Attenuation

**Fig. 5:**
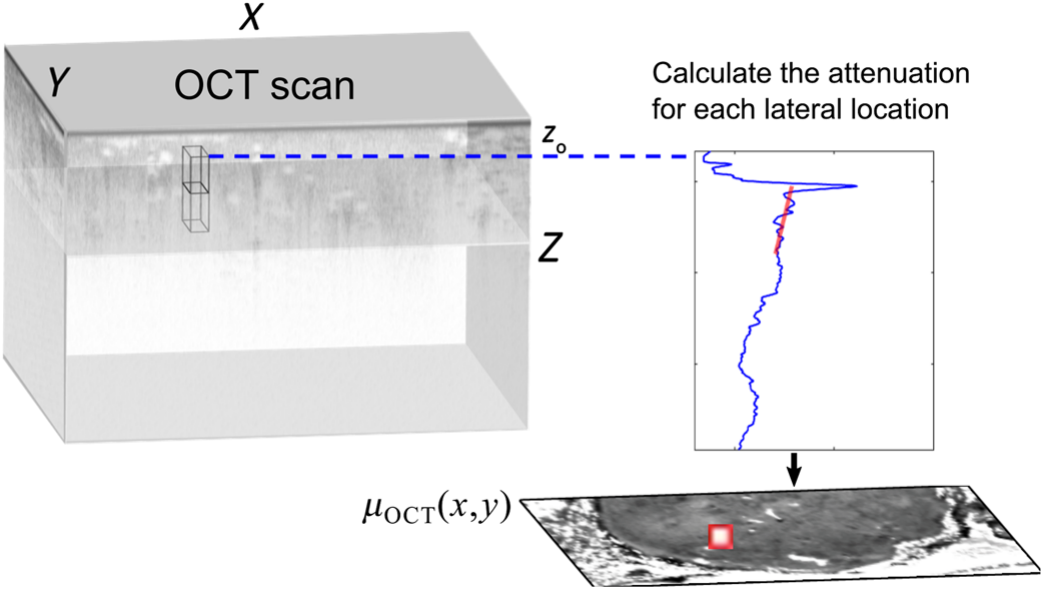
Schematic of OCT parametric attenuation coefficient imaging. The A-scan at each lateral location is averaged in a lateral (x–y) window outlined by the cuboid and used to calculate the attenuation coefficient in a depth (z) window, leading to a 2-D map in the *en face* (x–y) plane. Courtesy of Blake R. Klyen (unpublished).

OCT attenuation has been increasingly used for tissue characterization to provide additional contrast to the OCT structural information. Methods of analysis have made use of individual or multiple A-scans/B-scans or of volumetric scans to map the 2-D distribution of the attenuation as *en face* attenuation images, as shown in Fig. 5, which, in general terms, represents a form of parametric imaging.^8^^,^^9^ This section presents a survey of applications seeking to use OCT attenuation to characterize tissue, with a focus on skin, arteries, and tissues with cancer, including summarizing the characteristic attenuation coefficients of normal and diseased tissues. Overall, the results show promising examples of contrast between normal and diseased tissue. However, there are general issues with quantification evident in the range of experimentally reported values, which will be discussed in Sec. 5.

### 4.1 Skin: Dermatological Conditions and Burns

Cutaneous tissue in humans comprises a generally thin, superficial cellular epidermis overlaying a thicker layer of dermis containing various scatterers, including abundant collagen fibers. The OCT attenuation in the dermis has been assessed *in vivo*, but there is generally limited data in the epidermis,^1^ because on many parts of the body it is too thin (sub ∼100  μm for hairy skin) to be readily amenable to measurement. Schmitt et al.^1^ were the first to apply an OCT-based method (based on the single-scattering model) to measure the attenuation coefficient in normal cutaneous dermal tissue *in vivo*. They performed measurements at multiple body locations on two human subjects, including the forearm (mean $\mu_{\mathrm{OCT}}$: 4.6/4.7; AFR: 200 to 400  μm), finger (mean $\mu_{\mathrm{OCT}}$: 3.7/5.0; AFR: 250 to 500  μm), and lip (mean $\mu_{\mathrm{OCT}}$: $2.0\;{\mathrm{mm}}^{-1}$; AFR: 100 to 500  μm).

Later, Kholodnykh et al.^51^ studied and corrected the systematic errors in the measured attenuation coefficient caused by the CPSF. They applied their method to human forearm *in vivo*, reporting much higher attenuation values of dermis ($\mu_{\mathrm{OCT}}$: 10 to $13\;{\mathrm{mm}}^{-1}$; AFR: 100  μm) than Schmitt et al. at the same mean wavelength (1300 nm). They attributed this difference to the different experimental protocols, such as the pressure due to the contacting probe and the use of clearing agent (i.e., glycerol) in Schmitt et al.’s work. These factors will be further discussed in Sec. 5.

**Fig. 6:**
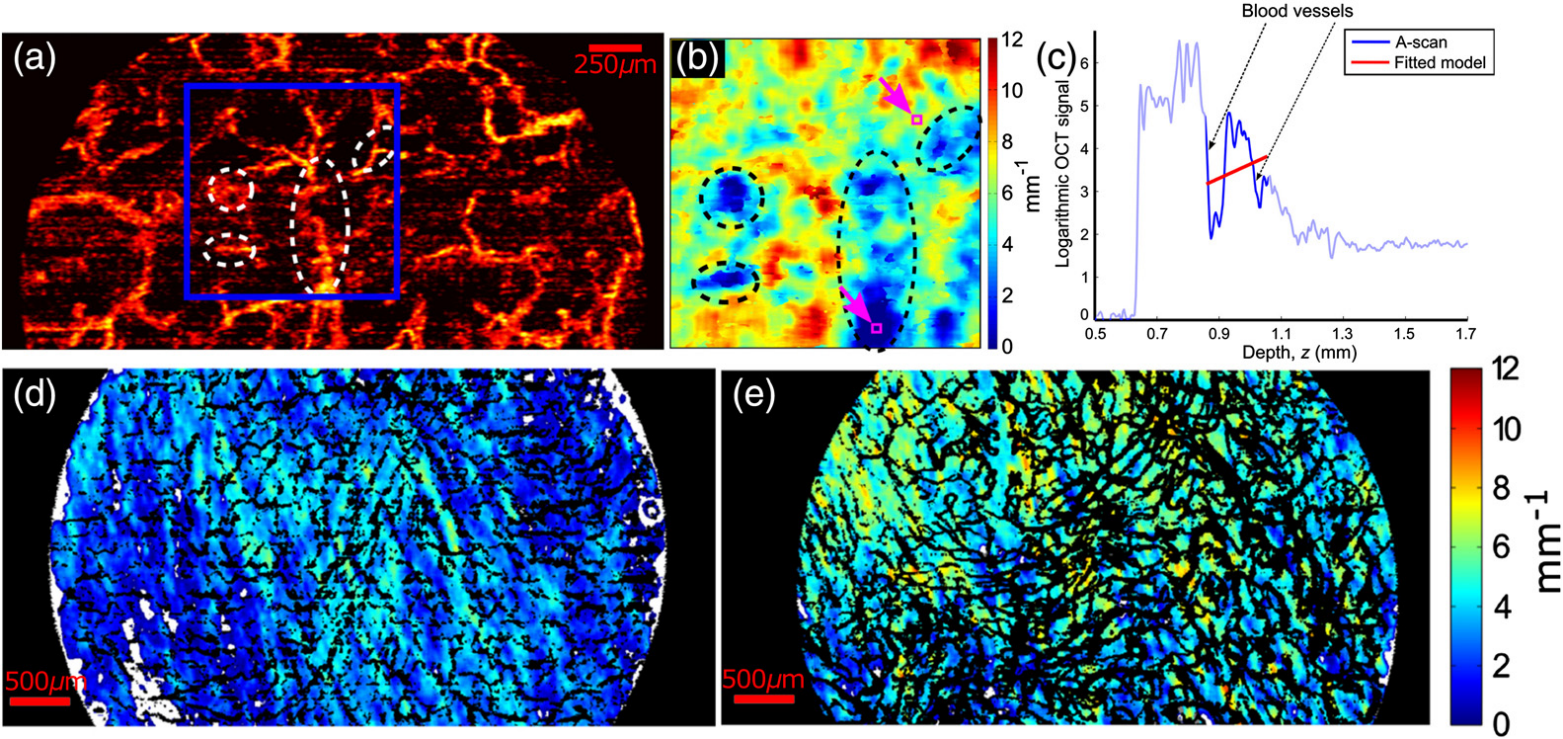
OCT attenuation imaging of human skin *in vivo*. (a) OCT vasculature image of normal skin. (b) Parametric attenuation coefficient imaging of the tissue region in the blue square in (a). Dashed circles outline the regions with incorrect attenuation coefficients due to the blood vessels. AFR is 200  μm with lateral averaging of 40×40  μm. (c) An example showing incorrect fitting caused by vessels, from the zone marked by the lower of the purple squares in (b). (d) and (e) Longitudinal parametric attenuation coefficient imaging of a human burn scar before and after laser treatment with vascular masks shown in black. AFR is 250  μm with lateral averaging of 20×20  μm. Adapted from Refs. 35 and 69.

The single-scattering model used in these studies assumes tissue homogeneity over the depth range (i.e., AFR) used to estimate the attenuation coefficient. However, the dermis is perfused with a network of blood vessels with highly distinct optical properties. Experimental determination of the OCT attenuation coefficient of whole blood is challenging due to the very high forward scattering of red blood cells.^47^ Bosschaart et al.^68^ modeled scattering properties of whole blood using Mie theory (to describe a single blood cell) combined with the Percus–Yevick structure factor to account for nonlinear scaling of optical coefficients with volume fraction, which are especially prevalent at high hematocrits. At 1300 nm, they found a high scattering coefficient of $35\;{\mathrm{mm}}^{-1}$ (close to values reported from the literature of $40\;{\mathrm{mm}}^{-1}$ at 1300 nm^68^) and a scattering anisotropy (g) of 0.96, indicating a very high degree of forward scattering by whole blood. These extreme values for blood compared with average values above in the 2- to $13\text{-}{\mathrm{mm}}^{-1}$ range highlight the distinct lack of homogeneity of the dermis. Additionally, many conditions are characterized by visible redness of the skin, indicating higher levels of blood than in healthy skin. This inhomogeneity leads to artifacts in the estimated attenuation coefficients when a vessel is contained in the fitting window, as shown in Figs. 6(b) and 6(c). Appreciation of this issue has only fairly recently been highlighted.^35^ Such artifacts may cause either underestimation or overestimation of the attenuation, depending on the size of the fitting window and its depth position relative to the vessels.

To mitigate this obvious source of inhomogeneity, Gong et al. presented a method to identify and mask the blood vessels from the attenuation estimation, using OCT speckle decorrelation for their detection,^70^^,^^71^ and provided parametric imaging of the attenuation coefficient of the remaining tissue.^35^ The resulting mean attenuation coefficient of dermis from normal human subjects (n=6) is $6.3\pm 0.5\;{\mathrm{mm}}^{-1}$ (AFR: 200  μm). Another distinction of their work is the use of a polarization-sensitive OCT (PS-OCT) scanner to mitigate the possible errors in the quantified attenuation coefficients due to the birefringence of dermal collagen, measured to be 0.4 to $1.3\times 10^{-3}$ by Gong et al.^72^ at 1325-nm wavelength and 0.5 to $1.1\times 10^{-3}$ by Pierce et al.^73^ at 1300-nm wavelength.

Using the attenuation coefficient of normal dermis as the baseline, OCT attenuation has been applied to the assessment of cutaneous conditions, such as psoriasis, which is characterized by patches of abnormal (often flaky and red) skin. Welzel et al.^74^ demonstrated a lower attenuation coefficient ($\mu_{\mathrm{OCT}}$: $2.9\pm 0.9\;{\mathrm{mm}}^{-1}$; AFR: not given; n=28) of psoriasis than in normal human skin ($\mu_{\mathrm{OCT}}$: $3.6\pm 1.5\;{\mathrm{mm}}^{-1}$; AFR: not given; n=28) in the upper dermis at 1300-nm wavelength. Their longitudinal monitoring further indicated an increase of the OCT attenuation in psoriasis after therapy ($\mu_{\mathrm{OCT}}$: $3.8\pm 1.7\;{\mathrm{mm}}^{-1}$; AFR: not given; n=17), approaching the measured normal skin attenuation ($\mu_{\mathrm{OCT}}$: $4.2\pm 1.6\;{\mathrm{mm}}^{-1}$; AFR: not given). They believe these characteristic attenuation coefficients are associated with inflammation in psoriasis, which can impact the density and distribution of collagen fibers and, thus, the scattering properties of the dermis. The impact of the presence of vasculature on these results is unknown.

Another example of the application of OCT attenuation measurement to cutaneous conditions is the assessment of human burn scars. Burns arise from various causes and lead to scarring, which presents as the proliferation of collagen and blood vessels in pathological scarring, including hypertrophic scars and keloids. In contrast to pathological scars, normotrophic scars present similar characteristics to the surrounding normal skin and represent the best clinical endpoint. To investigate the optical properties of burn scars, Gong et al.^35^ quantified the OCT attenuation of dermis with masking of blood vessels, providing parametric images. They found significantly lower values (hypertrophic scar $\mu_{\mathrm{OCT}}$: $3.8\pm 0.4\;{\mathrm{mm}}^{-1}$; normotrophic scar $\mu_{\mathrm{OCT}}$: $4.2\pm 0.9\;{\mathrm{mm}}^{-1}$; AFR: 200  μm) than those of the contralateral or adjacent normal skin ($\mu_{\mathrm{OCT}}$: $6.3\pm 0.5\;{\mathrm{mm}}^{-1}$; AFR: 200  μm; n=6), using a PS-OCT scanner. They attributed this difference to the reduced scattering in scar tissue arising from the higher water content and supported this assertion with corresponding optical propagation simulations showing a similar trend.

Es’haghian et al.^69^ further extended vasculature-masked OCT attenuation imaging to longitudinal monitoring of hypertrophic scars undergoing fractional laser ablation treatment, as shown in Figs. 6(d) and 6(e). They reported characteristic changes in the scar attenuation after treatment: an increase (31%±27%) and decrease (13%±5%) in the attenuation coefficient, respectively, in immature and mature scars; there was minimal change in the higher OCT attenuation coefficient ($\mu_{\mathrm{OCT}}$: $5.1\pm 0.7\;{\mathrm{mm}}^{-1}$; AFR: 250  μm) of the normal untreated skin (n=7). The difference in the average measured attenuation coefficient of normal skin from that estimated by Gong et al.^35^ could be due to many factors, including intersubject variation in skin type, variation in skin locations, the use of different AFRs and different instruments, and the use of PS-OCT. The longitudinal measurement/imaging of OCT attenuation provides an important approach for monitoring the tissue response to treatment. Assuming careful calibration is performed at each time point of measurement/imaging, longitudinal imaging potentially provides a useful relative measure of alterations over time, but more research is required to understand the meaning and reliability of absolute attenuation coefficients of skin.

There is also preliminary use of OCT attenuation to analyze cancer tissue in skin, which will be summarized in Sec. 4.3 on oncology.^15^^,^^64^ Additionally, Olsen et al.^75^ used OCT attenuation as a surrogate measure of skin edema, reporting an increase of the attenuation coefficient in edema of 10 subjects (median $\mu_{\mathrm{OCT}}$ increased from $1.8\;{\mathrm{mm}}^{-1}$ at baseline to $2.3\;{\mathrm{mm}}^{-1}$).

Overall, the characteristic attenuation coefficients of normal skin and skin conditions (Table 1), and their changes during treatment, indicate the great potential of OCT attenuation for clinical monitoring of skin conditions. The reported values in Table 1 show variations among different body locations, possibly caused by different tissue microstructures, and also for the same body locations, such as the normal forearm skin. This might be due to the variation between subjects and, more importantly, the inconsistency in data acquisition and processing methods. Such variations suggest the need for a standardization of methods in future to allow better comparison between studies.

**Table 1:** Summary of published values of OCT attenuation coefficient of human dermis *in vivo*. All results were calculated using single-scattering model.

Cutaneous tissue	Reference	Wavelength (nm)	AFR (μm)	Correction		Location	Sample number	Attenuation (${\mathrm{mm}}^{-1}$)
				CPSF	SRF			
Normal skin	Schmitt et al.^1^	1300	200 to 400	Y	N/A	Forearm	2 subjects	4.6/4.7 (mean)
			250 to 500			Finger		3.7/5.0 (mean)
			100 to 500			Lip		2.0 (mean)
	Kholodnykh et al.^51^	1300	100	Y	N/A	Forearm	NS	10 to 13
	**Gong et al.**^35^	1325	200	Y	Y	Forearm, thigh and lower leg	6 patients	6.3±0.5
	**Es’haghian et al.**^69^	1300	250	Y	Y	Upper arm, abdomen, back, thigh and calf	7 patients	5.1±0.7
	Welzel et al.^74^	1300	NS	NS	NS	Including forearm	28 patients	3.6 to 4.2 (mean)
Psoriasis	Welzel et al.^74^	1300	NS	NS	NS	Including forearm	28 patients (17 after treatment)	2.9±0.9 (untreated)
								3.8±1.7 (treated)
Burn scar	**Gong et al.**^35^	1300	200	Y	Y	Forearm, thigh and lower leg	6 patients	3.8±0.4 (hypertrophic)
								4.2±0.9 (normotrophic)

Note: Papers highlighted in bold present parametric attenuation coefficient imaging; others represent point measurements. AFR, axial fitting range; CPSF, confocal point spread function; N/A, not applicable; NS, not specified; SRF, sensitivity roll-off function.

### 4.2 Cardiology

**Fig. 7:**
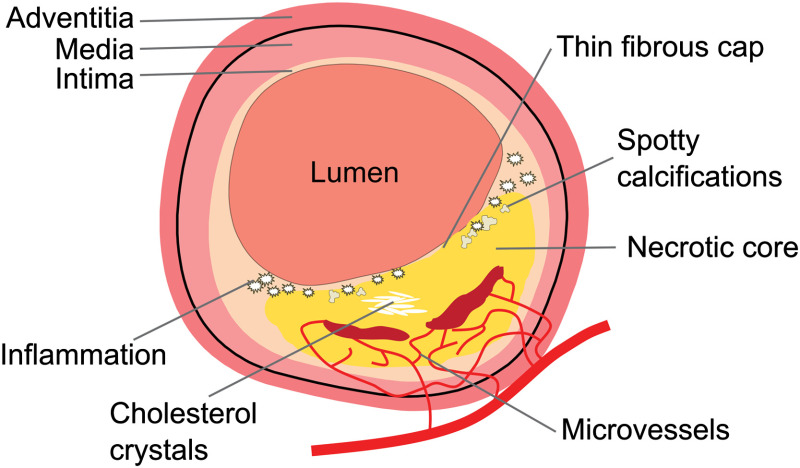
Schematic of the vessel wall with an advanced atherosclerotic plaque. The healthy wall consists of three layers (top): the intima, lying directly beneath the endothelium, the media, which consists of SMCs, and the adventitia, which is made up of connective tissues. These layers are separated by elastic membranes (not shown). A large thin-cap fibroatheroma (bottom) is a heterogeneous structure that exhibits a number of characteristics that may be recognized in OCT images and affect the attenuation coefficient. Adapted from Ref. 76.

The arterial system can be affected by atherosclerosis, a systemic inflammatory disease that gives rise to focal formations of fatty deposits in the vessel wall. This is a problem, in particular, in the carotid and coronary arteries, where disruption of those “plaques” can trigger thromboembolism, leading to ischemia in the brain (stroke) or heart muscle (myocardial infarction, i.e., heart attack). Catheter-based intravascular OCT is a powerful method for visualization of atherosclerosis and is routinely applied in guidance of minimally invasive coronary interventions. The normal structure of healthy coronary arterial wall consists of three layers: (1) a thin, bright intimal layer; (2) a darker medial layer with a thickness of 200 to 300  μm, consisting of smooth muscle cells (SMCs); and (3) the connective adventitia, which has a signal-rich and heterogeneous appearance on OCT. An example may be seen in Fig. 7 (top region). Atherosclerotic plaques form in the intimal layer, which thickens under the influence of the deposition of cholesterol and related compounds. Accumulation of these species triggers an inflammatory response, which leads to hypoxic conditions and subsequent necrosis. Detection of these lipid-rich necrotic cores in atherosclerotic plaque potentially enables pre-emptive interventions by medication or stenting. Quantitative characterization of the different tissue types, including calcification and fibrous tissues, is a potentially important application of OCT attenuation imaging.

Quantitative OCT analysis of atherosclerosis was first explored by Levitz et al.,^77^ who demonstrated that there is quantitative OCT attenuation contrast in different *ex vivo* arterial tissue components. Two studies by van der Meer et al.^7^^,^^78^ showed that such information could be extracted locally and, thus, be used for differentiating tissue types. The single-scattering model was applied to OCT data acquired from carotid arteries (n=13) *ex vivo* with an 800-nm time-domain scanner to quantify the attenuation of various tissue constituents.^78^ Differences between tissue types (lipid-rich, fibrous intimal thickening, calcification, and thrombus) were attributed to the different scatterers in these tissues, such as the highly scattering red blood cells, which lead to a high attenuation in the thrombi. These results were extended by imaging data of atherosclerosis with a time-domain system at a wavelength of around 1300 nm.^7^ They demonstrated the feasibility of using OCT attenuation to differentiate tissue types. Angle dependence of scattering parameters was investigated by Xu et al.,^8^ who demonstrated a strong dependence on imaging orientation for the highly oriented SMCs in the tunica media.

**Fig. 8:**
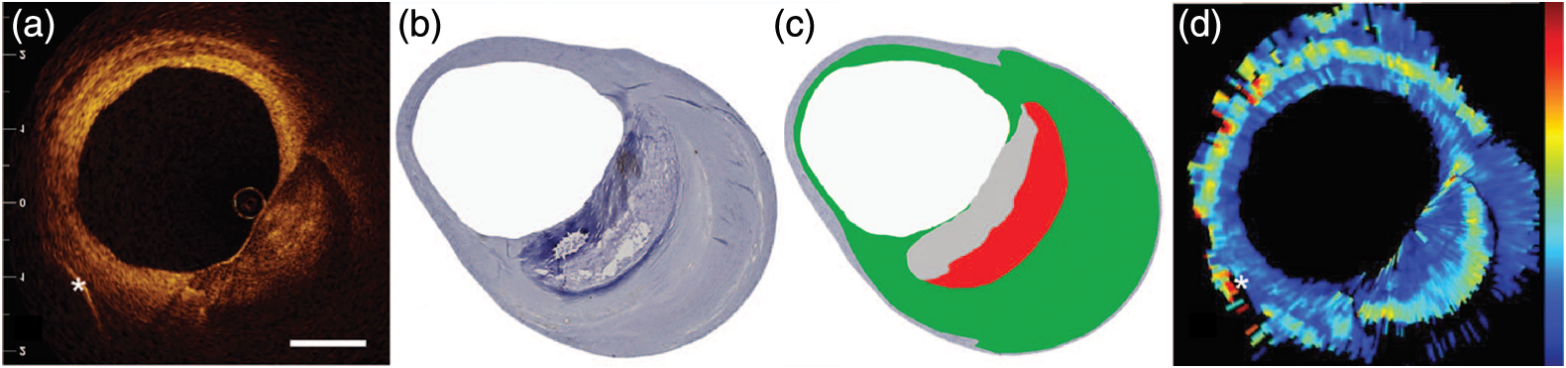
OCT attenuation imaging of a coronary artery with an atherosclerotic lesion *in vitro*. (a) and (b) OCT image and corresponding histology of the artery. (c) Cartoon overlaid on the histology to indicate an advanced necrotic core (red) behind a calcification (gray), and a slight fibrotic (green) circumferential intimal thickening. (d) OCT attenuation coefficient image ranging from 0 (blue) to $15\;{\mathrm{mm}}^{-1}$ (red). Scale bar: 1 mm. Adapted from Ref. 16.

Van Soest et al.^16^ further implemented OCT attenuation imaging of coronary arteries (n=65) in a catheter-based OCT system, approximating *in vivo* clinical imaging of coronary arteries. They demonstrated the differentiation of necrotic core and macrophage infiltration ($\mu_{\mathrm{OCT}}\geq 10\;{\mathrm{mm}}^{-1}$) from calcific and fibrous arterial tissue ($\mu_{\mathrm{OCT}}$: 2 to $5\;{\mathrm{mm}}^{-1}$) using the OCT attenuation (AFR: ≥200  μm). *Ex vivo* data were acquired with a time-domain OCT system. Figure 8 shows an example of a coronary atherosclerotic lesion with a necrotic core behind a calcified region, identified from histology [Fig. 8(b)] and marked in red in Fig. 8(c).^16^ The necrotic core and calcified region exhibit similar signal strengths in the structural OCT image in Fig. 8(a). Aided by OCT attenuation imaging in Fig. 8(d), the necrotic core is better contrasted with the calcified region than in the original OCT image. *In vivo* data from this study, recorded with a prototype swept-source OCT scanner, showed qualitatively and quantitatively similar attenuation patterns. These results illustrate the promise of OCT attenuation to complement the qualitative arterial tissue classification that relies on interpretation of image texture and structural features for determination of tissue composition and plaque type. Table 2 summarizes the quantified attenuation coefficients of various tissue types.

**Table 2:** Summary of published values of OCT attenuation coefficient of arterial tissue *ex vivo*. All results were calculated using single-scattering model except those by Liu et al. using the depth-resolved method.^40^

Arterial tissue	Reference	Wavelength (nm)	AFR (μm)	Correction		Location	Attenuation (${\mathrm{mm}}^{-1}$)
				CPSF	SRF		
Intimal thickening/fibrous	van der Meer et al.^78^	800	NS	Y	N/A	Carotid	5.5±1.2
	van der Meer et al.^7^	1300	NS	Y	N/A	NS	3.2±1.2
	**Xu et al.**^8^	1320	NS	Y	N/A	Coronary	6.4±1.2
	**van Soest et al.**^16^	1310	≥200	Y	Y	Coronary	2-5
	**Liu et al.**^40^	1310	N/A	N	N	Coronary	1.8±0.5
Lipid-rich region	van der Meer et al.^78^	800	NS	Y	N/A	Carotid	3.2±1.1
	van der Meer et al.^7^	1300	NS	Y	N/A	NS	2.3±0.5
	**Xu et al.**^8^	1320	NS	Y	N/A	Coronary	13.7±4.5
	**van Soest et al.**^16^	1310	≥200	Y	Y	Coronary	≥10
	**Liu et al.**^40^	1310	N/A	N	N	Coronary	2.6±0.1
Calcification	van der Meer et al.^78^	800	NS	Y	N/A	Carotid	11.1±4.9
	van der Meer et al.^7^	1300	NS	Y	N/A	NS	26±3.2
	**Xu et al.**^8^	1320	NS	Y	N/A	Coronary	5.7±1.4
	**Liu et al.**^40^	1310	N/A	N	N	Coronary	0.9±0.2
Macrophage infiltration	**van Soest et al.**^16^	1310	≥200	Y	Y	Coronary	>12
	**Liu et al.**^40^	1310	N/A	N	N	Coronary	3.4±0.4
Thrombus	van der Meer et al.^78^	800	NS	Y	N/A	Carotid	11.2±2.3
	Kume et al.^81^	1300	NS	N	NS	Coronary	3.8±1.0 (red)^a^
							2.1±0.3 (white)^a^

Note: Papers highlighted in bold present parametric attenuation coefficient imaging. AFR, axial fitting range; CPSF, confocal point spread function; N/A, not applicable; NS, not specified; SRF, sensitivity roll-off function.

a Measured in the data presented by the authors.

The attenuation coefficients vary significantly between the different studies reported in Table 2. Qualitative identification of vascular tissue has generally followed the classification of Yabushita et al.:^79^ fibrous tissue is homogeneous and signal-rich; calcified tissue is signal-poor with well-defined borders; and lipid-rich/necrotic tissue is signal-poor with diffuse borders. This set of criteria implicates low attenuation for both fibrous and calcified tissues, with high backscattering for fibrous tissue and low for calcifications. Based on a tissue optics interpretation of the qualitative classification, lipid-rich/necrotic tissue can be inferred to have strong attenuation. The attenuation (and backscattering, when provided) values reported by Xu et al.,^8^ van Soest et al.,^16^ and Liu et al.^40^ are consistent with this pattern; whereas, the contrast measured by van der Meer et al.^7^^,^^78^ appears to be inverted. A possible explanation of this difference may lie in the selection of fitting regions, which in the case of van der Meer et al. appears to exclude the signal-rich proximal areas in attenuating tissues, causing them to derive data from the slowly varying multiple-scattering background. The $\mu_{\mathrm{OCT}}$ values for different arterial tissue types do not significantly depend on temperature or tissue fixation, which eases the requirements on *ex vivo* studies.^80^

More recently, Liu et al. implemented a depth-resolved method on intravascular OCT scans acquired *in vitro* on 135 images from coronary arteries on two cadaver hearts.^40^ Using a variety of signal descriptors (intensity, attenuation, and backscatter), they were able to distinguish up to six different tissue types (mixed, calcified, fibrous, lipid-rich, macrophages, and necrotic core). Table 2 shows that the attenuation coefficient values reported by Liu et al.^40^ are much smaller than those reported by other studies.^8^^,^^16^ Two possible causes of this discrepancy are the blind reconstruction of amplitude (not intensity) data from 8-bit images stored on the acquisition system, which may introduce an unknown scale factor; and the omission of correction factors for CPSF or sensitivity roll-off. They also reported maximum and 95th percentile values for the parameters that they computed. These quantifiers, for the top of the distribution, are in good agreement with the values reported by Xu et al.^8^ and van Soest et al.^16^ The attenuation values computed by the depth-resolved model are expected to be affected by OCT speckle (see Sec. 3), but in general the effect of speckle filtering on this relation has not been studied. In the present case, the data was filtered post-hoc by application of a median filter, but the quantitative implications of this operation are unknown.

Gnanadesigan et al.^82^ derived relations between $\mu_{\mathrm{OCT}}$ and lipid-rich atherosclerotic plaque based on optimal classification accuracy in a receiver operating characteristic analysis. They showed that, with histology control, lipid-core plaque has $\mu_{\mathrm{OCT}}>8.5\;{\mathrm{mm}}^{-1}$. Thin-cap fibroatheroma, as identified in clinical OCT images, was found to have $\mu_{\mathrm{OCT}}>11\;{\mathrm{mm}}^{-1}$.^83^ Their approach to comprehensive validation of 3-D data sets, rather than matching of individual OCT images to histology, enables statistically robust analyses with minimal operator bias.

**Fig. 9:**
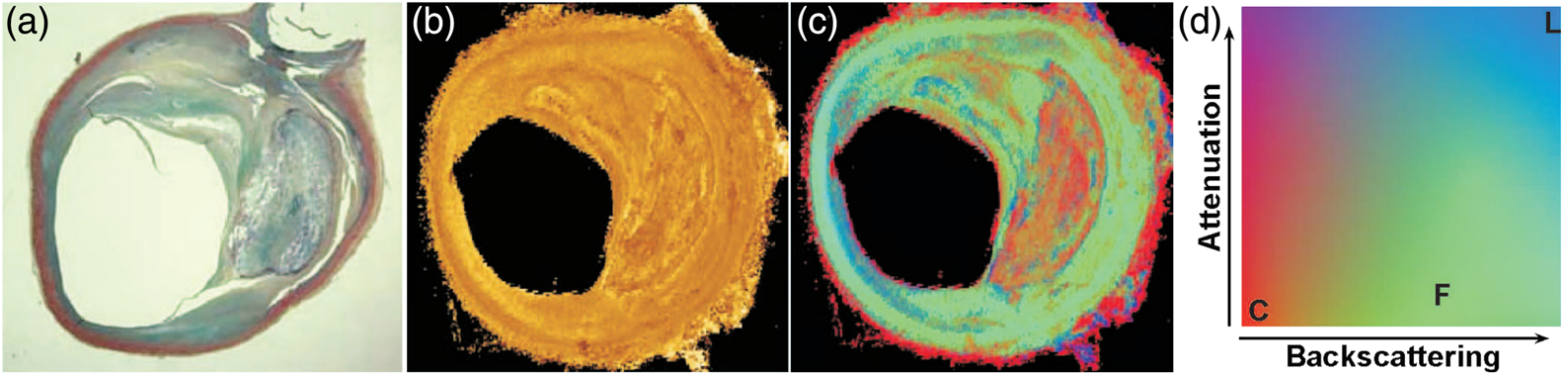
Combined imaging of OCT attenuation and backscattering coefficient of a fibrocalcific plaque. (a) and (b) Images of histology and OCT attenuation coefficient. (c) Image of the combined attenuation and backscattering coefficient using the colormap in (d). C, calcific tissue; F, fibrous tissue; L, lipid tissue. The three tissue types led, respectively, to attenuation coefficients of $5.7\pm 1.4\;{\mathrm{mm}}^{-1}$, $6.4\pm 1.2\;{\mathrm{mm}}^{-1}$, and $13.7\pm 4.5\;{\mathrm{mm}}^{-1}$; and backscattering coefficients of $4.9\pm 1.5\;{\mathrm{mm}}^{-1}$, $18.6\pm 6.4\;{\mathrm{mm}}^{-1}$, and $28.2\pm 8.9\;{\mathrm{mm}}^{-1}$, respectively. Adapted from Ref. 8.

When different arterial tissue types present similar attenuation properties, tissue characterization with OCT attenuation coefficient alone is ineffective. A combination of the OCT attenuation with additional OCT-derived optical properties by Xu et al.^8^ and Liu et al.^40^ resulted in statistically significant discrimination between tissues types. An example from Xu et al. is shown in Fig. 9, where the calcific (red) and fibrous (green) tissues are better differentiated using the combined attenuation and backscattering coefficient image in Fig. 9(c) than using only the attenuation in Fig. 9(b). Such combination of multiple parameters provides one promising approach to enhance tissue contrast and, thus, may provide better tissue classification for future applications, if they can be reliably extracted from catheter-based measurements.

### 4.3 Oncology

**Fig. 10:**
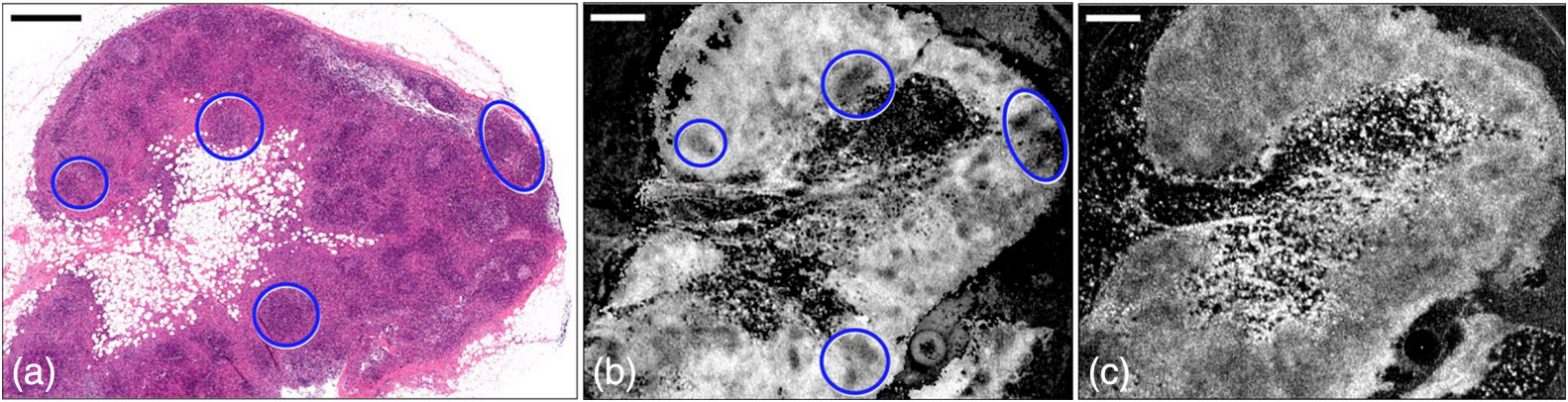
OCT attenuation imaging of a malignant human axillary lymph node. (a)–(c) Histology, OCT attenuation, and structural OCT image of the lymph node. The circles highlight the residual, noncancerous cortical tissue. Scale bars: 1 mm. Adapted from Ref. 9.

Another promising application of OCT attenuation imaging is in the characterization of cancer. Expected changes in tissue caused by cancer, relevant to OCT, include altered cellular arrangement; density and size of nuclei and organelles; proliferation and changes in the organization of the extracellular matrix; and changes in the blood and lymphatic microvasculature.^84^^,^^85^ McLaughlin et al.^9^ were the first to apply parametric OCT attenuation imaging to assess cancer *ex vivo*, extracting an attenuation coefficient for each A-scan and visualizing the spatially distributed attenuation values as an *en face* image (Fig. 10). Although they quantified only the relative values of attenuation coefficient, the demonstration on malignant human axillary lymph nodes from breast cancer patients (n=2) indicated the presence of contrast between malignant and healthy non-neoplastic tissue. Figure 10 shows one such example indicating the differentiation of residual healthy tissue (circled regions) in a malignant lymph node as the low attenuation coefficient regions in Fig. 10(b), which is difficult to identify using the original OCT image in Fig. 10(c). This contrast was attributed to the changes in size and texture of cell nuclei resulting from the neoplastic transformation.^86^ Their method was further developed and applied by Scolaro et al.^17^ for imaging the absolute attenuation coefficients in axillary lymph nodes (n=4). They summarized the OCT attenuation coefficients of various tissue subtypes, as included in Table 3, to guide the classification of different tissue types within the lymph node. A strikingly attractive feature of the results of that work is the relative lack of overlap between tissue types and attenuation coefficient values—a fact that would need further testing given the small sample size.

**Table 3:** Summary of published values of OCT attenuation coefficient of human cancers for various tissue and cancer types. Results were calculated using the single-scattering model. All results were acquired *ex vivo* except those from the skin cancer by Wessels et al.,^14^ UC by Bus et al.,^3^^,^^88^ and brain tissue by Almasian et al.^93^

Tissue	Paper	Tissue type	Sample number	Wavelength (nm)	AFR (μm)	Correction		Attenuation (${\mathrm{mm}}^{-1}$)
						CPSF	SRF	
Axillary lymph nodes in breast cancer	**Scolaro et al.**^17^	Paracortex	4 axillary lymph nodes	1320	200	Y	N/A^a^	10.0 to 11.5
		Primary inactive cortical follicles						4.5 to 6.8
		Medullary sinus						8.1 to 9.1
		Fibrous capsule						14.1 to 15.3
		Thickened fibrous capsule						11.6 to 12.6
		Necrotic						13.0 to 14.5
		Dystrophic calcification						16.0 to 16.5
Ovarian cancer	Yang et al.^13^^,^^87^	Normal	51 ovaries	1310	NS	Y	N	2.41±0.59^13^/2.38±0.67^87^
		Malignant						1.55±0.46^13^/1.74±0.55^87^
UC	Cauberg et al.^18^	Benign	54 bladder samples from 18 patients	850	>100	N/A^b^	N/A	5.75 (median)
		UC						5.52, 4.85 and 5.62 (median for grade 1, 2 and 3 UC)
	Bus et al.^3^	Grade 2 UC	29 lesions	1310	>100	N	N	1.97 (IQR: 1.57 to 2.30)
		Grade 3 UC	13 lesions					3.52 (IQR: 2.74 to 3.94)
	Bus et al.^88^	Low grade UC	26 patients	1310	>100	N	N	2.1 (IQR: 1.7 to 2.3)
		High grade UC						3.0 (IQR: 2.9 to 3.7)
	Freund et al.^89^	Low grade UC	35 patients	1310	>100	Y	Y	3.3 (IQR: 2.7 to 3.7)
		High grade UC						4.9 (IQR: 4.3 to 6.1)
Prostate cancer	Muller et al.^90^^,^^95^	Benign	20 patients in Ref. 95	1310	500	Y	Y	3.8 (mean)^90^/4.6 (median)^95^
		Malignant						4.1 (mean)^90^/5.0 (median)^95^
	**Swaan et al.**^96^	9 subtypes	1 patient	1310	320	Y	Y	4.0 to 7.0 (mean)
Brain cancer	**Kut et al.** for white matter analysis^92^	Noncancer white matter of seizure patients	16 patients in the training group	1310	NS	Y	Y^c^	6.2±0.8
		Infiltrated zone						2.7±1.0 for low-grade (LG)
								3.5±0.8 for high-grade (HG)
		Cancer core						4.0±1.4 for LG
								3.9±1.6 for HG
	**Kut et al.** for gray matter analysis^92^	Noncancer gray matter of seizure patients	32 patients in the training and validation group					2.8±0.9
		Infiltrated zone						3.6±1.4 for LG
								3.7±1.2 for HG
		Cancer core						3.8±1.2 for LG
								4.2±1.5 for HG
	Almasian et al.^93^	Cortical tissue	5 patients	1300	200	Y	Y	3.8±1.2 (normal) versus 3.6±1.1 (glioma)
		Subcortical tissue	3 patients					5.7±2.1 (normal) versus 4.5±1.6 (glioma)
	**Yashin et al.**^94^	Normal white matter	30 patients	1300	NS	N	N	8.5 (IQR: 8.2 to 9.3)
		Normal cortex						5.0 (IQR: 3.2 to 5.5)
		Astrocytoma grade I to III						3.0 (IQR: 2.6 to 3.5)
		Glioblastoma grade IV without necrotic areas						3.2 (IQR: 2.6 to 4.2)
		Glioblastoma grade IV with necrotic areas						6.3 (IQR: 5.4 to 6.8)
		Necrosis						7.5 (IQR: 5.3 to 7.7)
Kidney cancer	Barwari et al.^97^^,^^98^	Normal	14 patients^97^/16 cases^98^	1300	NS	Y	Y	4.95^97^/5.0^98^ (median)
		Renal cell carcinoma						8.86^97^/8.2^98^ (median)
	Wagstaff et al.^99^	Oncocytoma	40 patients	1310	NS	Y	Y	3.4 (median)
		Renal cell carcinoma						4.4/4.8 (median)
	Buijs et al.^91^	Benign	95 patients	NS	NS	Y	Y	3.2 (IQR: 2.7 to 4.4)
		Renal cell carcinoma						4.3 (IQR: 3.7 to 5.0)
Skin cancer	Wessels et al.^14^	Benign lesions	33 patients	1300	NS	Y	Y	5.49±0.24
		Melanoma						4.28±0.44
	Wessels et al.^15^	Healthy skin	20 lesions of 16 patients	1300	NS	Y	Y	2.1±1.4
		Vulvar intraepithelial neoplasia						6.2±2.1

Note: Papers highlighted in bold present parametric attenuation coefficient imaging. AFR, axial fitting range; CPSF, confocal point spread function; IQR, interquartile range; N/A, not applicable; NS, not specified; SRF, sensitivity roll-off function.

a The depth scan response in the time-domain OCT scanner was corrected.

b Dynamic focusing was used.

c Sensitivity fall-off was corrected together with the confocal function using the phantom signal.

OCT attenuation has also been used to characterize ovarian cancer, which is difficult to diagnose at an early stage due to the lack of symptoms, resulting in the lowest survival rate of the gynecologic cancers. Yang et al.^13^ applied OCT attenuation to ovaries (n=18) *ex vivo*, showing lower attenuation values in malignant tissue ($\mu_{\mathrm{OCT}}$: $1.6\pm 0.5\;{\mathrm{mm}}^{-1}$) than in normal tissue ($\mu_{\mathrm{OCT}}$: $2.4\pm 0.6\;{\mathrm{mm}}^{-1}$). In a subsequent study, they demonstrated consistent contrast between the malignant ($\mu_{\mathrm{OCT}}$: $1.7\pm 0.6\;{\mathrm{mm}}^{-1}$) and normal tissue ($\mu_{\mathrm{OCT}}$: $2.4\pm 0.7\;{\mathrm{mm}}^{-1}$) in ovaries (normal: n=26; malignant: n=7),^87^ and further augmented the attenuation with measurement of the cumulative phase retardation caused by the birefringence of collagen tissue. The combination of these two parameters shows high sensitivity and specificity for the classification of malignant and normal ovary tissue. They further explained the influence of the measured collagen area fraction lower in the malignant than in the normal tissue on the contribution of the collagen tissue to the two quantified optical properties.

Analysis of urothelial carcinoma (UC) by OCT attenuation has also been investigated. Cauberg et al.^18^ measured OCT attenuation in human bladder samples (n=54) to assist grading of UC tissue *ex vivo*. They reported the attenuation coefficients (overspecified to two decimal places) for different tissues, including benign tissue (median $\mu_{\mathrm{OCT}}$: $5.75\;{\mathrm{mm}}^{-1}$); and grade 1 (median $\mu_{\mathrm{OCT}}$: $5.52\;{\mathrm{mm}}^{-1}$), 2 (median $\mu_{\mathrm{OCT}}$: $4.85\;{\mathrm{mm}}^{-1}$), and 3 (median $\mu_{\mathrm{OCT}}$: $5.62\;{\mathrm{mm}}^{-1}$) UC at 850-nm wavelength. Bus et al.^3^ extended this analysis to patients (n=7) *in vivo* and reported the higher attenuation in grade 3 (median $\mu_{\mathrm{OCT}}$: $3.53\;{\mathrm{mm}}^{-1}$) than 2 (median $\mu_{\mathrm{OCT}}$: $1.97\;{\mathrm{mm}}^{-1}$) lesions. A follow-on study on a large number of patients (n=26) by Bus et al. further validated the higher attenuation of high-grade lesions (median $\mu_{\mathrm{OCT}}$: $3.0\;{\mathrm{mm}}^{-1}$) than low-grade lesions (median $\mu_{\mathrm{OCT}}$: $2.1\;{\mathrm{mm}}^{-1}$), demonstrating the feasibility of OCT attenuation for grading low- and high-grade lesions.^88^ A recent study on 35 patients by Freund et al.^89^ demonstrated a consistent contrast in OCT attenuation, as summarized in Table 3.

OCT attenuation has also been applied to investigate other types of cancer. For example, Muller et al.^90^ used OCT attenuation to aid in the characterization of prostate cancer, reporting attenuation coefficients of benign (mean $\mu_{\mathrm{OCT}}$: $3.8\;{\mathrm{mm}}^{-1}$; AFR: 500  μm) and malignant (mean $\mu_{\mathrm{OCT}}$: $4.1\;{\mathrm{mm}}^{-1}$; AFR: 500  μm) tissue. They noted that not all tissue with high attenuation coefficient corresponded to cancer. Buijs et al.^91^ explored the use of OCT attenuation to assist the differentiation of renal cell carcinoma (median $\mu_{\mathrm{OCT}}$: $4.3\;{\mathrm{mm}}^{-1}$), respectively, versus benign renal masses (median $\mu_{\mathrm{OCT}}$: $3.2\;{\mathrm{mm}}^{-1}$) and versus oncocytomas (median $\mu_{\mathrm{OCT}}$: $3.4\;{\mathrm{mm}}^{-1}$), based on 95 patients. The results showed the improved diagnostic yield aided by OCT attenuation.^91^ Kut et al.^92^ investigated the OCT attenuation of cancerous and noncancerous brain tissue from human patients (n=37) and reported lower attenuation coefficients in cancerous tissue (infiltrated zone mean $\mu_{\mathrm{OCT}}$: 3.5 and $2.7\;{\mathrm{mm}}^{-1}$, cancer core mean $\mu_{\mathrm{OCT}}$: 3.9 and $4.0\;{\mathrm{mm}}^{-1}$, respectively, for high-grade and low-grade) than in noncancerous white matter tissue (mean $\mu_{\mathrm{OCT}}$: $6.2\;{\mathrm{mm}}^{-1}$). They attributed this contrast to the invading cancer cells, which break down and decrease the expression of myelin in white matter. Similar contrast was recently reported by Almasian et al.^93^ from an *in vivo* study, as summarized in Table 3. A recent study on *ex vivo* human brain tissue by Yashin et al.^94^ reported consistently lower attenuation coefficients of the tumorous tissue than the normal tissue. In addition, they observed a significant influence of tumor necrosis on the measured attenuation coefficients. Wessels et al.^15^ applied OCT attenuation analysis to vulvar intraepithelial neoplasia (VIN), which can progress to vulvar squamous cell carcinoma. The results showed higher attenuation coefficients in VIN ($\mu_{\mathrm{OCT}}$: $6.2\pm 2.1\;{\mathrm{mm}}^{-1}$) than in healthy skin ($\mu_{\mathrm{OCT}}$: $2.1\pm 1.4\;{\mathrm{mm}}^{-1}$). They also investigated cutaneous melanoma and reported lower attenuation in melanomas (mean $\mu_{\mathrm{OCT}}$: $4.3\;{\mathrm{mm}}^{-1}$) than in benign lesions (mean $\mu_{\mathrm{OCT}}$: $5.5\;{\mathrm{mm}}^{-1}$).^14^ The results in these various pilot studies indicate the general promise of OCT attenuation as a biomarker to characterize cancer.

### 4.4 Other Tissues

Building on from the wide application of OCT to ophthalmology, OCT attenuation analysis has been explored to investigate eye diseases *in vivo*, such as glaucoma. The OCT attenuation in the retinal nerve fiber layer (RNFL) of healthy (n=10 with an RNFL thickness ∼50 to 220  μm) and glaucomatous subjects (n=30) has been analyzed by van der Schoot et al.,^100^ showing decreasing values with severity in glaucoma (overspecified to 2 decimal places): (mild glaucoma $\mu_{\mathrm{OCT}}$: $4.09\pm 0.34\;{\mathrm{mm}}^{-1}$; moderate glaucoma $\mu_{\mathrm{OCT}}$: $3.14\pm 0.22\;{\mathrm{mm}}^{-1}$; and advanced glaucoma $\mu_{\mathrm{OCT}}$: $2.93\pm 0.33\;{\mathrm{mm}}^{-1}$), as compared to normal subjects ($\mu_{\mathrm{OCT}}$: $4.78\pm 0.46\;{\mathrm{mm}}^{-1}$). Consistent contrast was later presented by Vermeer et al. in a study on healthy (n=10) and glaucomatous (n=8) eyes.^101^ They explained this contrast as due to the decreased density of nerve fibers in glaucoma. Additionally, the OCT attenuation coefficients of the multiple tissue layers (including the RNFL) in the retina have been extracted by DeBuc et al.^102^ and Sun et al.^103^ to assess other diseases that cause changes in the retina, including diabetes and pituitary adenoma. However, despite the promise, larger sample sizes are needed to establish the feasibility of OCT attenuation for assessing these diseases.

In additional, OCT attenuation has been applied to the characterization of various other tissue pathologies, such as parametric imaging of dystrophic muscle tissue, to identify necrotic lesions in mouse models of muscular dystrophy (necrotic lesion $\mu_{\mathrm{OCT}}$: $9.6\pm 0.3\;{\mathrm{mm}}^{-1}$ and necrotic myofiber $\mu_{\mathrm{OCT}}$: $7.0\pm 0.6\;{\mathrm{mm}}^{-1}$ versus healthy tissue $\mu_{\mathrm{OCT}}$: $3.9\pm 0.2\;{\mathrm{mm}}^{-1}$; AFR: 500  μm),^104^ and measurement of cartilage tissue to quantify differences between the healthy cartilage ($\mu_{\mathrm{OCT}}$: $9.7\pm 3.3\;{\mathrm{mm}}^{-1}$), repaired tissue ($\mu_{\mathrm{OCT}}$: $3.1\pm 1.4\;{\mathrm{mm}}^{-1}$), and bone ($\mu_{\mathrm{OCT}}$: $4.5\pm 0.5\;{\mathrm{mm}}^{-1}$) in goats with induced osteochondral defects.^105^ All of these studies on OCT attenuation, both at the level of initial demonstration and relatively large-scale clinical data sets, have shown good potential for improved quantitative tissue characterization as compared to the use of only the qualitative OCT images.

## 5 Discussion

### 5.1 Measurement Variation, Accuracy, and Precision

The use of OCT attenuation for tissue characterization and differentiation is gradually expanding, and promising examples of contrast between normal and diseased tissue have been shown.^9^^,^^13^^–^^19^ However, there are overall large variations in the attenuation values of the same tissue types across studies, as summarized in Sec. 4. There are general issues with OCT attenuation measurement, making it challenging to fully understand the origins of such large variations. These issues include the lack of clarity of the instrument configuration, data processing method, depth range for attenuation calculation, accuracy, and precision (e.g., attenuation coefficients presented with too many significant figures), and inconsistency in how the values are reported (median versus mean versus value range). Therefore, we urge authors of future studies to clearly specify the details of the implementation of OCT attenuation calculations to provide improved clarity and consistency, including at least the following.

1. **Instrument and measurement configuration**: OCT scanner type, wavelength, imaging resolutions (axial, including assumptions on refractive index, and lateral), sampling density, use of polarization diversity detection, and contact/noncontact scanning mode.
2. **Data processing key parameter and method**: OCT model, preprocessing (e.g., averaging and tissue surface detection), correction for CPSF and sensitivity roll-off, axial range for fitting/calculation, fitting/calculation method and assessment of fit quality, masking of vessels for *in vivo* scans and processing time.
3. **Sample handling and results presentation**: provide assessment of measurement accuracy and precision, report the attenuation coefficient range, mean, median, and variation, and specify the number of samples and measurements within samples, tissue locations, and tissue processing if any (e.g., time from harvesting, freezing, optical clearing and fixing/histological tissue processing).

Reporting the above will improve the comparability, and ultimately reproducibility, of results across laboratories and enable understanding of the current variations as well as point to solutions.

The accuracy and precision of OCT attenuation measurement have not been comprehensively investigated in most studies, which in part have contributed to the large variations. To date, the most practical approach to assess the accuracy and precision is using a homogeneous imaging phantom with well controlled and known optical properties, such as polystyrene microsphere or silica bead solutions. The theoretical attenuation coefficients are estimated using a scattering model, such as Mie theory, for a set of phantoms with varying concentrations (or even sizes) of scatterers and, thus, varying attenuations. The measured OCT attenuation of the phantoms can then be compared to the theoretical values, building a calibration curve for estimating the accuracy and precision, as performed by Almasian et al.^28^

One issue with this approach is the reduced accuracy when the concentrations of the scatterers are sufficiently high,^28^ for appreciable “multiparticle scattering” (i.e., interferences between the densely distributed scatterers) to occur.^106^ To mitigate this, the estimation of the theoretical attenuation coefficient with discrete particle models must be augmented to take into account such effects. In addition, the approach involves the preparation of multiple solutions of scatterers, which is tedious but feasible. A solid phantom with structured attenuation (e.g., an array of thin pillars filled with medium with varying attenuations) is highly desirable and can be feasible based upon progress in imaging phantom fabrication achieved to date.^107^^,^^108^ Such a phantom could then be replicated and be readily applied in multiple studies for multi-laboratory validation, which would provide an important assessment of measurement accuracy and precision and help understand and minimize the large variations that currently exist. In addition, it is also possible to assess the accuracy and precision by comparing OCT attenuation to other methods for measuring tissue optical properties, such as measurement of light transmittance through the sample,^109^ but this approach has not been explored so far.

### 5.2 Tissue Heterogeneity

Biological tissue typically presents heterogeneity along both the axial and lateral directions in the OCT imaging volume. The lateral heterogeneity in tissue is less problematic than the axial heterogeneity, and in fact favorable when parametric imaging of the tissue attenuation is required. The axial heterogeneity complicates OCT attenuation measurement in particular as one crucial assumption in the OCT models is homogeneity over the depth range used in the calculation.

Image segmentation, thus, forms an important part of an automated workflow for OCT attenuation analysis, so as to restrict the depth range for calculation to relatively homogeneous tissue regions. The first step in such segmentation is detecting the tissue surface, which can avoid artificial nonphysical negative attenuation resulting from the inclusion of noise signal above the tissue surface, as highlighted by Yuan et al.^36^ There are multiple methods available for surface detection, mainly using the markedly strong reflectance and correspondingly high OCT signal at the tissue surface. These methods include the use of the local maximum OCT signal intensity, local maximum gradient of the OCT signal, and the Canny edge detector.^70^^,^^110^^–^^112^ The underlying tissue may present different morphologies and need further segmentation, especially when the tissue presents layered structures with varying OCT signal strength. For example, normal skin comprises epidermis (low signal) and dermis (high signal), and normal coronary arterial wall comprises intima (high signal), media (low signal), and adventitia (high signal), which are further segmented to confine the depth ranges for attenuation measurement.^112^^,^^113^ Overall, the capacity to locate homogeneous tissue regions for attenuation calculation is dependent on the specific tissue morphology and the corresponding contrast in OCT signal and may need additional revision of the methods under specific disease conditions. Such revisions will be disease- and tissue-dependent and may not be feasible for all conditions.

The impact of axial tissue heterogeneity can also be mitigated by data processing methods in the single-scattering model, using the depth-resolved method or an adaptive window fitting approach. The depth-resolved method calculates the attenuation coefficient for each tissue depth (local attenuation coefficient) and does not require tissue homogeneity on the scale (100 to 500  μm) required by the fitting method.^38^ However, as it assumes the full attenuation of light in each A-scan, the obtained attenuation coefficients are sensitive to the estimation of the noise level and, thereby, likely to be less reliable in cases where contributions from multiple scattering are significant. Additionally, the correction of system-dependent functions (i.e., CPSF and sensitivity roll-off) and noise in this approach have not yet been fully described.^41^ As well, it is not yet clear how to assess the accuracy of the volumetric attenuation coefficients obtained using this method. Further research is still needed to address these issues. On the other hand, adaptive window fitting extends the model fitting to variable depth ranges in each A-scan, by tuning the length of the fitting window from a fixed start depth or the depth of the window with fixed length, to generate parametric volumes.^16^^,^^17^ In these implementations, the goodness of fit is calculated for each fitting and used to select the optimal fitting range and the resulting attenuation coefficient. One disadvantage is the significantly longer computation time due to the increased number of fits performed. Care should also be taken to validate if the optimal fitting leads to a physically reasonable attenuation coefficient. Apparently good fitting may sometimes lead to an artificial nonphysical negative attenuation coefficient when the fitting extends from a locally low signal region to a deeper region with high signal.

Speckle averaging provides an important method to mitigate the impact of the local heterogeneity and the inherent speckles on attenuation measurement. Even in homogeneous samples, the presence of OCT speckles requires the use of averaging in the axial and/or lateral directions to provide reliable attenuation estimation. Although speckle averaging/reduction is an active field of research,^114^ there is a need for further study to assess the impacts of the methods and degree of averaging on the estimated attenuation coefficients and to suggest optimal averaging.

One ubiquitous source of tissue heterogeneity in living tissue is the microvascular network, comprising blood and lymphatic vessels.^115^ Each type of vessel presents very different scattering properties to those of the surrounding tissue, creating local heterogeneity in the OCT signal that has rarely been taken into account. The blood vessels present very high levels of scattering, which at the OCT wavelengths is strongly forward directed due to the large, high-contrast scatterers (mainly red blood cells).^116^ This contrast leads to lower OCT signals in vessel regions than in the surrounding tissue and artifacts in the estimated attenuation coefficients when the calculation window covers the vessel pixels, as demonstrated by Gong et al.^35^ The lymphatic vessels have been observed first by Vakoc et al.^115^ to present even lower signals than the blood vessels, almost approaching the OCT noise floor, due to the transparency (i.e., absence of scatterers) of the lymph.^117^^–^^119^ This transparency, which has also been observed in nerve fibers,^120^ can lead to similar artifacts in attenuation analysis. One approach to eliminate such artifacts is segmenting the vessels with OCT angiography and/or lymphangiography, and masking them to restrict the attenuation analysis to A-scans without vessels.^35^ In addition, such implementation can also provide a more comprehensive tissue characterization, namely using OCT attenuation and vascular imaging to assess the avascular tissue components and the microvascular network, respectively.

### 5.3 Other Factors Affecting Measurement

Multiple additional factors can impact the measurement of OCT attenuation, including the pressure induced by contact scanning, tissue birefringence, and the use of optical clearing agents. Contact scanning mode is used either intentionally, such as for skin imaging to mitigate motion artifact, or unintentionally in intravascular imaging due to the uncontrolled positioning of the imaging catheter inside the vessels. It is well known that compression of a sample induces more scattering. This phenomenon is largely the simple consequence of increasing the gradient of the refractive index by reducing the axial dimensions between scatterers. This pressure-enhanced scattering is captured by the OCT signal and can then alter the measured attenuation, but its quantitative effect on the OCT attenuation coefficient has not been studied. Interestingly, Kholodnykh et al.^51^ reported approximately twice the OCT attenuation value of that measured by Schmitt et al.^1^ from human forearm skin at the same OCT wavelength (1300 nm) and attributed this difference, in part, to the pressure resulting from the contacting probe. As a comparison, the pressure-induced changes to the reduced scattering coefficient have been extensively studied in fiber-probe-based diffuse reflectance spectroscopy. For example, Reif et al.^121^ reported an increase of the reduced scattering coefficient at 700 nm with the increasing pressure, which was attributed to the increased density of the scatterers, although there are also inconsistent results in the field.^122^ Quantification of the impacts of pressure on OCT attenuation is still needed, and the use of contact scanning needs to be clarified in future studies.

Many tissues are birefringent due to the presence of long thin fibrous structures, such as the abundant collagen fibers in dermal skin with locally or globally unidirectional arrangement. The interactions of birefringent tissue with OCT light leads not only to attenuation but also to variation in the polarization state of light with tissue depth.^123^ An attenuation measurement of a birefringent sample with a conventional OCT system can be problematic, as the detected OCT reflectance signal strength depends not only on the tissue attenuation but also on the polarization state of the incident light, the OCT components that can alter the light polarization, and the birefringence of the tissue sample. For example, the logarithmic OCT structural B-scans of birefringent samples can show a banding-like pattern versus depth in tissue, which modulates the exponential decay expected in the single-scattering model for homogeneous tissue regions. Such dependence of the OCT signal on the tissue birefringence will then lead to inaccuracy of the estimated attenuation coefficients. Simultaneous detection of the OCT signal in two orthogonal polarization channels can eliminate such polarization artifacts.

Optical clearing is a widely studied method of introducing an agent to make tissue more transparent to allow greater light penetration and provide consequently deeper imaging.^124^ The scattering in tissue originates from gradients caused by mismatches in the refractive index of scatterers. The main contributors to the gradients, as modeled by Schmitt and Kumar,^125^ include tissue fibers (bundles of elastin and collagen), cytoplasmic organelles (e.g., mitochondria), and cell nuclei, in contrast to the cytoplasm and interstitial fluid with lower refractive index. Optical clearing agents are thought to penetrate into extracellular spaces and reduce the mismatch in refractive index. This then leads to reduced scattering (i.e., elevated tissue transparency) and enhanced imaging depth in tissue.^124^ As a result, optical clearing has been shown to reduce attenuation coefficients in the OCT signal. For example, Deng et al.^126^ investigated OCT attenuation of rat skin *in vivo* following the application of polyethylene glycol with a penetration enhancer. They reported a decrease of the attenuation from 7.0 to $4.9\;{\mathrm{mm}}^{-1}$ at 120 min after the application of glycerol. Genina et al.^127^ reported a decrease of OCT attenuation by values in the range 16% to 32% for different optical clearing agents applied to rat dermis. Measurement of the OCT attenuation provides a method to assess the optical clearing effects.^126^^,^^127^ Notwithstanding its long gestation, further work is needed to understand the utility of optical clearing in practical applications.

### 5.4 Complementary Optical Properties

To enhance the contrast for tissue characterization and differentiation, additional optical properties can be obtained from the OCT signal to supplement attenuation, such as the backscattering coefficient ($\mu_{b,\mathrm{NA}}$) (or local reflectance used by Levitz et al.^54^). The backscattering coefficient of the sample, detected within the NA of the OCT system, has been extracted from the OCT amplitude after correcting for system parameters.^8^^,^^28^^,^^54^^,^^128^^–^^131^ Xu et al.^8^ and Liu et al.,^40^ respectively, added the backscattering coefficient and backscatter term (linearly related to the logarithm of the backscatter coefficient) to the attenuation coefficient, providing an approach to further differentiate atherosclerotic tissue components with similar attenuation coefficients.

The simultaneous measurement of attenuation and backscattering coefficients also allows the extraction of further parameters, including the anisotropy and the size of the scatterers. Kodach et al.^128^ calculated the ratio of backscattering coefficient to the total scattering coefficient (extracted from the OCT signal) as the phase function integrated over the NA ($p_{\mathrm{NA}}$). They then built two calibration functions, including $p_{\mathrm{NA}}$ versus particle diameter and anisotropy versus particle diameter, aided by Mie theory. The OCT-estimated $p_{\mathrm{NA}}$ of an Intralipid sample was input into the $p_{\mathrm{NA}}$ versus particle diameter function to decide the scatterer size, which was further input into the anisotropy versus particle diameter function to decide the anisotropy. A similar approach has been used by Schneider et al.^129^ for measuring the size of dispersed polystyrene nanoparticles. Levitz et al.^54^^,^^130^ proposed the simultaneous calculation of attenuation and local reflectance, which were then fed into the calibration grid in the attenuation–local reflectance map to extract the anisotropy of developing collagen gels. To our knowledge, these methods for estimating the scatterer anisotropy and size have not yet been demonstrated on biological samples.

As described above, PS-OCT detects the polarization states of the reflected OCT light, aided by polarization diversity detection.^123^ Estimation of the birefringence and related polarization parameters (e.g., degree of polarization uniformity and optic axis orientation) represents an interesting alternative form of parametric imaging both with versions that integrate over similar axial regions as OCT attenuation imaging^72^^,^^132^ and local versions.^133^^,^^134^ Finally, retrieval of diattenuation—the polarization state-dependent differential attenuation—is also possible via PS-OCT but to the authors’ knowledge is thought to be a small effect and has not yet been widely investigated.^135^^,^^136^ Also based on polarization detection, Yashin et al.^94^ recently used cross-polarization OCT to simultaneously measure the attenuation coefficients of the co- and cross-polarized detected light. Demonstrations on human and rat brain tissues have indicated the potential for differentiation of cancer and normal tissue.^94^^,^^137^

We finally observe that, as well as optical properties, parametric OCT imaging can also be used to observe mechanical properties of tissue.^138^^,^^139^ All such properties, optical and otherwise, show great potential to be combined with OCT attenuation imaging to provide a more comprehensive assessment of tissues.

## 6 Conclusion

For more than 25 years, OCT has been studied and used as a tool for characterizing morphology on the 1- to 15-μm resolution scale. At the same time, it is widely accepted that submicrometer microscopic changes in tissue structure and organization due to disease progression causes altered tissue optical properties, which can possibly be probed by the OCT attenuation coefficient. Multiple models have been advanced to obtain the attenuation coefficient, ranging in sophistication, but by far the most commonly used model is the single-scattering model, which is used in almost all studies on clinically relevant tissues reported in this review. Our overview of these studies highlights the generally small sample sizes both on *ex vivo* and *in vivo* samples. The reported values of the OCT attenuation coefficient suggest that, although most pathologies show a change in $\mu_{\mathrm{OCT}}$, the difference between normal and diseased tissue is not always significant. In some studies, relative $\mu_{\mathrm{OCT}}$ values alone are sufficient, whereas other studies show the effectiveness of combining $\mu_{\mathrm{OCT}}$ with other OCT-derived parameters in differentiating healthy and diseased tissues. Our overview also highlights the wide spread in reported $\mu_{\mathrm{OCT}}$ values, which results from the use of different systems, methodologies, and sample preparation, as well as on occasions a lack of rigor in approach. Thus, whilst we remain optimistic overall, efforts toward standardization on a rigorous methodology in future research are crucial to advancing this field.
